# Transitioning from Methanol to Olefins (MTO) toward a Tandem CO_2_ Hydrogenation Process: On the Role and Fate of Heteroatoms (Mg, Si) in MAPO-18 Zeotypes

**DOI:** 10.1021/jacsau.3c00768

**Published:** 2024-02-13

**Authors:** Tomás Cordero-Lanzac, Izar Capel Berdiell, Alessia Airi, Sang-Ho Chung, Jenna L. Mancuso, Evgeniy A. Redekop, Claudia Fabris, Leidy Figueroa-Quintero, Juan C. Navarro de Miguel, Javier Narciso, Enrique V. Ramos-Fernandez, Stian Svelle, Veronique Van Speybroeck, Javier Ruiz-Martínez, Silvia Bordiga, Unni Olsbye

**Affiliations:** † Department of Chemistry, SMN Centre for Materials Science and Nanotechnology, University of Oslo, 0371 Oslo, Norway; ‡ Department of Chemistry, NIS Center and INSTM Reference Center, University of Turin, Turin 10125, Italy; § KAUST Catalysis Center (KCC), King Abdullah University of Science and Technology, Thuwal 23955-6900, Saudi Arabia; ∥ Center for Molecular Modeling, Ghent University, Technologiepark 46, B-9052 Zwijnaarde, Belgium; ⊥ Inorganic Chemistry Department, Laboratory of Advanced Materials, University Materials Institute of Alicante, University of Alicante, Apartado 99, Alicante 03080, Spain

**Keywords:** CO_2_ valorization, MTH, MTO, MgAPO-18, AEI, deactivation

## Abstract

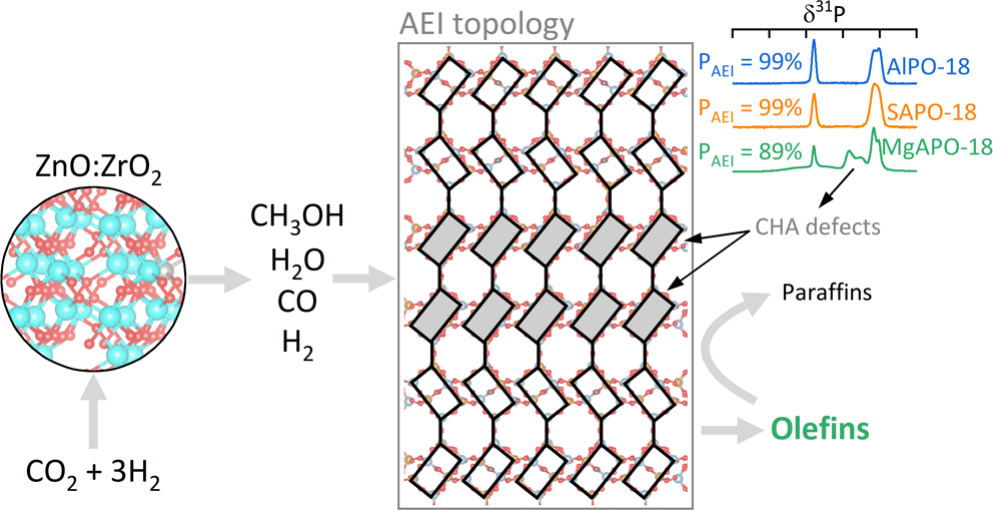

The tandem CO_2_ hydrogenation to hydrocarbons over mixed metal oxide/zeolite catalysts (OXZEO) is an efficient way of producing value-added hydrocarbons (platform chemicals and fuels) directly from CO_2_*via* methanol intermediate in a single reactor. In this contribution, two MAPO-18 zeotypes (M = Mg, Si) were tested and their performance was compared under methanol-to-olefins (MTO) conditions (350 °C, *P*_CH_3_OH_ = 0.04 bar, 6.5 g_CH_3_OH_ h^–1^ g^–1^), methanol/CO/H_2_ cofeed conditions (350 °C, *P*_CH_3_OH_/*P*_CO_/*P*_H_2__ = 1:7.3:21.7 bar, 2.5 g_CH_3_OH_ h^–1^ g^–1^), and tandem CO_2_ hydrogenation-to-olefin conditions (350 °C, *P*_CO_2__/*P*_H_2__ = 7.5:22.5 bar, 1.4–12.0 g_MAPO-18_ h mol_CO_2__^–1^). In the latter case, the zeotypes were mixed with a fixed amount of ZnO:ZrO_2_ catalyst, well-known for the conversion of CO_2_/H_2_ to methanol. Focus was set on the methanol conversion activity, product selectivity, and performance stability with time-on-stream. *In situ* and *ex situ* Fourier transform infrared spectroscopy (FT-IR), X-ray diffraction (XRD), solid-state nuclear magnetic resonance (NMR), sorption experiments, and *ab initio* molecular dynamics (AIMD) calculations were performed to correlate material performance with material characteristics. The catalytic tests demonstrated the better performance of MgAPO-18 versus SAPO-18 at MTO conditions, the much superior performance of MgAPO-18 under methanol/CO/H_2_ cofeeds, and yet the increasingly similar performance of the two materials under tandem conditions upon increasing the zeotype-to-oxide ratio in the tandem catalyst bed. *In situ* FT-IR measurements coupled with AIMD calculations revealed differences in the MTO initiation mechanism between the two materials. SAPO-18 promoted initial CO_2_ formation, indicative of a formaldehyde-based decarboxylation mechanism, while CO and ketene were the main constituents of the initiation pool in MgAPO-18, suggesting a decarbonylation mechanism. Under tandem CO_2_ hydrogenation conditions, the presence of high water concentrations and low methanol partial pressure in the reaction medium led to lower, and increasingly similar, methanol turnover frequencies for the zeotypes. Despite both MAPO-18 zeotypes showing signs of activity loss upon storage due to the interaction of the sites with ambient humidity, they presented a remarkable stability after reaching steady state under tandem reaction conditions and after steaming and regeneration cycles at high temperatures. Water adsorption experiments at room temperature confirmed this observation. The faster activity loss observed in the Mg version is assigned to its harder Mg^2+^-ion character and the higher concentration of CHA defects in the AEI structure, identified by solid-state NMR and XRD. The low stability of a MgAPO-34 zeotype (CHA structure) upon storage corroborated the relationship between CHA defects and instability.

## Introduction

CO_2_ capture and utilization (CCU) has potential as a key technology for the post-fossil society, where it will enable the production of fuels as well as consumer goods with properties equal to those obtained by converting fossil carbon. In this context, the conversion of CO_*x*_ (*x* = 1, 2) and hydrogen *via* oxygenates (methanol, dimethyl ether, ketene) to hydrocarbons over a tandem catalyst is a promising approach to produce specific ranges of hydrocarbons, such as BTX (benzene, toluene, and xylenes) for specialty chemicals, olefins for polyolefin production, as well as paraffins and alkane-aromatic mixtures for fuels.^1^ The tandem catalyst generally consists of a metal and/or metal oxide component to form the oxygenate and a zeolite/zeotype component to convert the oxygenate to hydrocarbons. This commonly named OXZEO catalyst achieves product distributions beyond the Fischer–Tropsch limitation,^2^ yet in a broader range to what is obtained in the conversion of methanol to hydrocarbons (MTH) over the same zeolite/zeotype.^3^ Some differences exist, which have been ascribed partly to the presence of additional components in the gas feed, in particular, high-pressure H_2_, CO, and H_2_O.^3−7^

An inherent challenge of the OXZEO process is that the conditions for the direct synthesis of hydrocarbons from CO_2_ are intermediate between the ideal conditions for methanol synthesis and methanol conversion, respectively. Importantly, this shift of conditions implies that zeotype catalyst performance and ranking may be different from those of the well-studied MTO process, hence opening a new arena for catalyst investigations. Even though the MTH-commercialized catalysts SAPO-34^8−10^ and H-ZSM-5^11,12^ have received most attention as the acidic function in the OXZEO catalyst, the comparison made by Su et al.^13^ between the former and its SAPO-18 counterpart suggested the potential of SAPO-18 (with the AEI framework) to outperform SAPO-34 (with the CHA framework) in the tandem process aimed at light olefin production. In essence, AEI and CHA are very similar topologies composed of the same layer units (double T6 rings connected along the diagonals) stacked with inversion symmetry (CHA) or a mirror plane (AEI) between neighboring layers. The authors ascribed the differences in activity to the weaker acidity generated in the AEI structure by the incorporation of the same amount of Si, which also significantly increases the barrier for hydrogen transfer and therefore improves the olefin/paraffin ratio. The same conclusion was reported by Zhang et al.^14^ in the tandem CO_2_ hydrogenation combining a ZnO:ZrO_2_ mixed oxide with SAPO-34 and -18. To study the unique effect of acid strength in the AEI topology, we recently introduced a series of MAPO-18 catalysts (M = Mg, Co, Zn, Si) and tested them in the conversion of methanol to light olefins (MTO), revealing that, indeed, the addition of H_2_ and CO to the methanol feed at high pressure had a stronger effect on materials containing divalent heteroatoms in the lattice compared to the one containing lattice Si. In particular, olefin hydrogenation and coke formation were more strongly suppressed in the M(II)APO-18 materials, leading to higher olefin/paraffin ratios and turnover numbers (TONs) for these materials.^15^ These results were intriguing, especially since the M(II)APO-18 materials have stronger Brønsted acid sites than SAPO-18, a property known to promote hydrogen transfer reactions and coke formation during the MTH reaction in zeolites/zeotypes.^16^ Furthermore, specifically in the tandem CO_*x*_ conversion, Li et al.^17^ demonstrated an enhanced activity for secondary reactions, including ethylene oligomerization and hydrogenation in SAPO-18 with the highest acid strength.

The strongly diffusion-limiting window size of MAPO-18 (3.8 Å × 3.8 Å) prevents mechanistic detail from being gained through effluent product analysis. However, low temperature and *in situ* FT-IR studies of the CoAPO-18 and SAPO-18 samples revealed that CoAPO-18 contained Brønsted acidic Co-(OH)-P bridge sites, as well as two Lewis acid sites: one associated with lattice Co and one associated with Co ion exchanged onto Brønsted acid sites. Ultimately, the CoAPO-18 catalyst quickly formed retained aromatic compounds during MTO, while the SAPO-18 catalyst predominantly formed alkenes in the first few minutes of the reaction, with a gradual buildup of aromatic compounds.^18^ Prior studies of MAPO-5 (M = Mg, Co, Zn, Si, Ti, Zr), a 1D open-channel 12-ring structure (AFI), revealed that a higher acid strength (M = Mg, Co, Zn) led to higher alkene selectivity compared to SAPO-5, which produced more aromatic compounds.^19^ Theoretical studies of propene versus benzene methylation reactions in MAPO-*X* structures (*X* = 5, 18, 34) suggested that the larger transition state (TS) from benzene methylation is better stabilized by the lattice than the propene methylation TS, leading to lower acid strength sensitivity for benzene than for propene methylation.^20−22^ This conclusion was supported by experimental studies of benzene versus propene methylation in MAPO-5 (M = Mg, Si, Zr). The experimental study further showed a higher selectivity toward hydrogen transfer reactions in MgAPO-5 than in SAPO-5.^23^ Similar effects of alkaline earth metals were previously observed by theoretical and experimental studies of H-ZSM-5, before and after ion exchange of M^2+^ (M = Ca, Sr, Mg) onto Brønsted acid sites.^24^

In this study, we investigated and compared MgAPO-18 and SAPO-18 as the zeotypes within the OXZEO catalyst. This was done in conjunction with a ZnO:ZrO_2_ mixed oxide, recognized for its high methanol selectivity and stability under the demanding tandem conditions at elevated temperatures.^8,11,14,25^ Kinetic studies, as well as material characterization using XRD, IR, solid-state NMR spectroscopy and *ab initio* molecular dynamics (AIMD) calculations, revealed details about the state of the zeotypes and how it affects their catalytic performance as the studied conditions gradually transitioned from the MTO *via* the methanol/CO/H_2_ cofeed to the CTO (CO_2_/H_2_-to-olefins) process. Special attention has been paid to the role of water in the reaction medium in this process transition, as well as the major role played by water in the fate of heteroatoms when the zeotypes are stored.

## Results and Discussion

### Role of Heteroatoms in MTO and CO_2_ Tandem Hydrogenation

**Figure 1:**
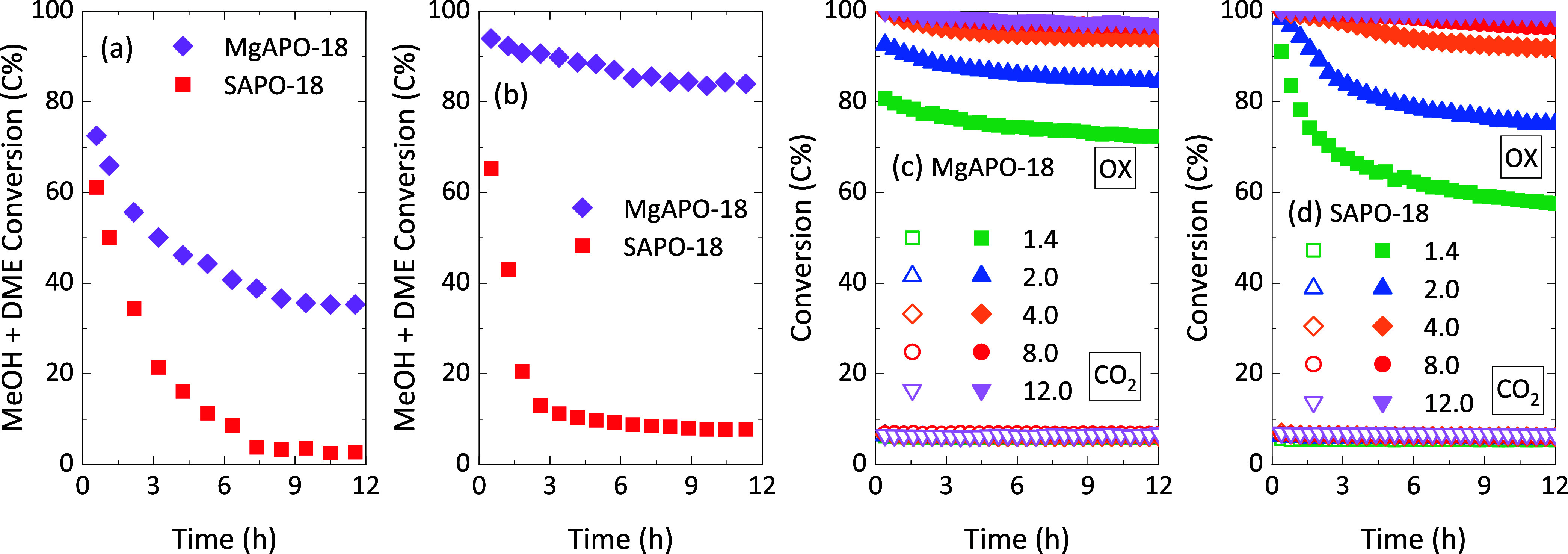
Evolution with time of oxygenate conversion in the methanol-to-olefins reaction using the MAPO-18 zeotypes (M = Mg, Si) at (a) 350 °C, 1 bar, 40 mbar methanol in He, 6.5 g_MeOH_ h^–1^ g^–1^ and (b) 350 °C, 30 bar, 1 bar methanol in H_2_/CO with a 3/1 ratio, 2.5 g_MeOH_ h^–1^ g^–1^. Evolution with time of CO_2_ and oxygenate (OX, methanol + DME) conversions in the CO_2_ tandem hydrogenation to hydrocarbons using ZnO:ZrO_2_ + (c) MgAPO-18 and (d) SAPO-18 catalysts at different MAPO-18 space times: 1.4 (green), 2.0 (blue), 4.0 (orange), 8.0 (red), and 12.0 g_MAPO-18_ h mol_CO_2__^–1^ (violet) at 350 °C, 30 bar, H_2_/CO_2_ ratio of 3, fixed CO_2_/ZnO:ZrO_2_ ratio. Samples were calcined and stored before testing.

Two MAPO-18 zeotypes, synthesized by the isomorphic substitution of Mg or Si in the AlPO-18 (AEI) framework, have been systematically compared. Both zeotypes were prepared following a similar synthesis protocol and show comparable crystallinity, Brunauer–Emmett–Teller (BET) specific surface area, and acidity (Figure S1 and Table S1). At MTO conditions (1 bar and methanol in N_2_ feed), steady conversion was reached by MgAPO-18, while SAPO-18 suffered complete deactivation with time on stream (Figure 1a). Furthermore, MgAPO-18 clearly outperformed the Si version of the zeotype when cofeeding methanol (1 bar partial pressure) with H_2_ and CO at 30 bar total pressure (Figure 1b). A more detailed study of these materials during MTO in the presence of CO, H_2_, and high pressure can be found in our previous work.^15^ Briefly, the study showed that high-pressure H_2_ addition led to higher turnover numbers before deactivation (TON) for both catalysts and also to hydrogenation of olefinic products to paraffins. Simultaneous introduction of CO, H_2_, and methanol inhibited olefin hydrogenation, and the sustained superior stability of MgAPO-18 was retained (although to a limited extent for SAPO-18).

When mixing these MAPO-18 zeotypes with ZnO:ZrO_2_, the one-step tandem hydrogenation of CO_2_ to hydrocarbons showed an enhanced performance compared to the hydrogenation of CO_2_ to methanol over the mixed ZnO:ZrO_2_ oxide (Figure S2a): using the same GHSV in terms of the oxide amount in the catalytic bed, the addition of MgAPO-18 increases the initial CO_2_ conversion by 2.3 points (all reaction indexes are defined in the Supporting Information). This goes hand in hand with a decrease in CO selectivity, which ultimately leads to increased hydrocarbon production compared to the methanol one. After 12 h on stream, the effect of tandem conditions is also noticeable (Figure S2b), and yet it has reached a steady state (Figure S2c). Notably, under conditions of accelerated deactivation at high GHSV and low MAPO-18 space time, the decline in oxygenate conversion for both MgAPO-18 and SAPO-18 zeotypes during tandem CO_2_ hydrogenation is quite similar (Figure 1c,d). Note that product distribution only undergoes a certain time evolution (Figure S2c). Moreover, the slight decrease in CO selectivity may indicate changes in the oxide function responsible for the hydrogenation of CO_2_, as observed for ZnO-containing catalysts.^26^ Consequently, the product distribution changes toward the steady state of the catalyst, producing slightly smaller amounts of CO and yielding an improved selectivity to hydrocarbons (Figure S2).

The presence of hydrogen has been identified to play a role in extending the lifetime of zeotypes with small windows such as SAPO-34.^4^ To evaluate the H_2_ effect on zeotype deactivation, experiments with different H_2_/CO_*x*_ ratios in the feed were carried out with and without feeding methanol. In the tandem CO_2_ hydrogenation using ZnO:ZrO_2_ and MgAPO-18, CO_2_ conversion increases with H_2_ partial pressure (Figure S3a). However, oxygenate conversion remains stable even with the lowest H_2_/CO_2_ feed (N_2_ was added as inert to maintain the GHSV). Please note that methanol and DME intermediates are steadily converted in the tandem CO_2_ hydrogenation with these H_2_/CO_*x*_ ratios. Otherwise, oxygenate conversion decayed slower when increasing the H_2_ partial pressure in reactions where H_2_ and a CO/CO_2_ mixture were cofed with methanol at 20 bar (Figure S3b). This is more significant in the case of MgAPO-18, with 30% higher oxygenate conversion when increasing the H_2_/CO_*x*_ ratio from 2/1 to 3/1.

Water is another molecule that is highly relevant for the comparison of catalyst performance between MTO conditions and the tandem conversion of CO_2_/H_2_ to olefins. Water competes with methanol and DME for adsorption at the Brønsted acid sites^27^ and may form protonated water clusters at high water contents, thereby slowing down methanol conversion.^28−30^ Considering the materials studied here, Valecillos et al.^31^ reported studies of an SAPO-18 catalyst where water addition to the methanol feed led to unselective quenching of the hydrocarbon pool species formation and conversion reactions due to competitive water adsorption on Brønsted acid sites. The resulting reduction of the relative reaction *vs* diffusion rates (Thiele modulus) may strongly contribute to coke formation mitigation. Another study suggested that hydrolysis of formaldehyde, which is a known coke precursor, might be the reason for the reduced coking rate observed with water addition.^32^

Considering next the concerted effect of methanol and water, Portillo et al.^33^ studied the effect of methanol and water on the deactivation of SAPO-34 by cofeeding these reaction intermediates with CO_2_ and H_2_. They concluded that the presence of water in the reaction medium lessened coking significantly at conditions of high H_2_ pressure, while increasing the partial pressure of methanol led to the opposite effect. The critical role of both methanol concentration and water presence is demonstrated here, with huge performance differences between MgAPO-18 and SAPO-18 catalysts at high methanol partial pressure and the absence of water on the one hand (Figure 1b) and similar performance at tandem CO_2_ hydrogenation conditions on the other hand (Figure 1c,d). The presence of water, produced in the conversion of CO_2_/H_2_ over the ZnO:ZrO_2_ function, along with the lower partial pressure of methanol in the reaction medium (formed and converted *in situ*), may be the reason for the similar performance of MgAPO-18 and SAPO-18 zeotypes due to reduction of methanol conversion rates and mitigation of coke formation caused by high water and low methanol partial pressures in the tandem catalyst bed. Potential back-mixing in the CO_2_ hydrogenation setup, due to the low flow required for meaningful conversion, will make the influence of water in the whole bed even stronger. In summary, when comparing deactivation rates during tandem CO_2_ hydrogenation, MgAPO-18 performs only slightly better. At the lowest MAPO-18 space time (1.4 g_MAPO-18_ h mol_CO_2__^–1^), C_2_–C_4_ hydrocarbon space time yields (STY) of 1.93 and 1.56 mol kg_MAPO-18_^–1^ (after 10 h on stream) were observed for the Mg and Si versions of the zeotype, respectively (Table 1), whereas huge differences were noticeable only in the direct conversion of methanol at 1 or 30 bar.

**Table 1:** C_2_–C_4_ Hydrocarbon Space Time Yield (STY) after 10 h On Stream in the CO_2_ Tandem Hydrogenation to Hydrocarbons and Methanol-to-Hydrocarbons

	C_2_–C_4_ STY (mol kg_MAPO-18_^–1^)	
	MgAPO-18	SAPO-18
CO_2_ hydrogenation at 30 bar^a^		
1.4 g_MAPO-18_ h mol_CO_2__^–1^	1.93	1.56
2.0 g_MAPO-18_ h mol_CO_2__^–1^	1.57	1.48
4.0 g_MAPO-18_ h mol_CO_2__^–1^	0.88	0.84
8.0 g_MAPO-18_ h mol_CO_2__^–1^	0.50	0.46
12.0 g_MAPO-18_ h mol_CO_2__^–1^	0.30	0.30
MTO at 1 bar	2.83	1.06
MTO at 30 bar cofeeding H_2_/CO	2.23	0.37

a The same CO_2_/ZnO:ZrO_2_ ratio was used in all cases to achieve comparable results.

**Figure 2:**
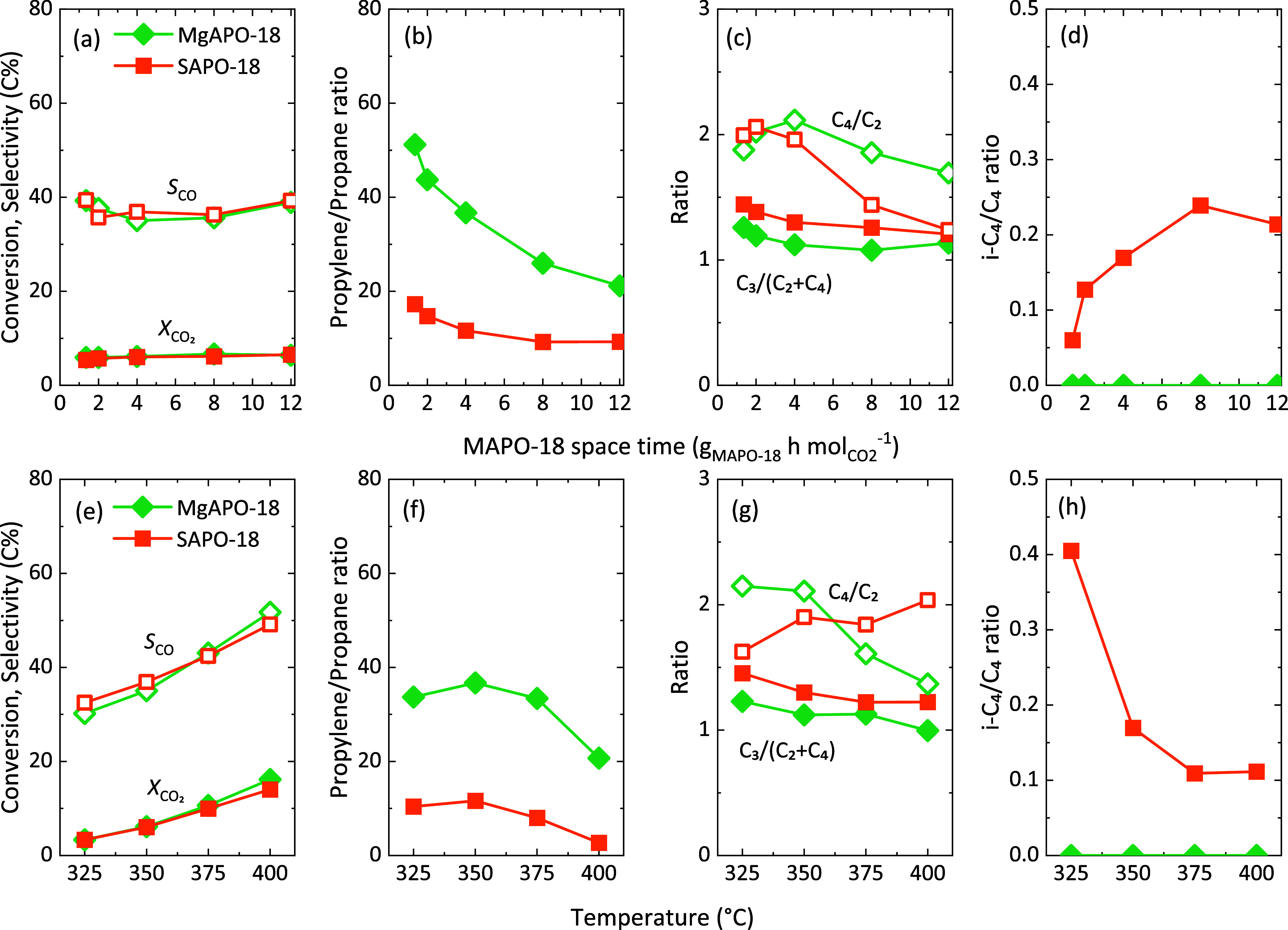
Effect of (a–d) MAPO-18 (M = Mg, Si) space time (at 350 °C) and (e–h) temperature (at 4 g_MAPO-18_ h g_CO_2__^–1^) on the steady-state (a, e) CO_2_ conversion and CO selectivity, (b, f) propylene/propane ratio, (c, g) C_4_/C_2_ and C_3_/(C_2_ + C_4_) ratios, and (d, h) *i*-C_4_/C_4_ ratio in the CO_2_ tandem hydrogenation to hydrocarbons using ZnO:ZrO_2_ + MgAPO-18 and SAPO-18 catalysts. 30 bar, H_2_/CO_2_ ratio of 3, fixed CO_2_/ZnO:ZrO_2_ ratio. Samples were calcined *ex situ* and stored before testing.

A systematic study on the influence of the main reaction variables was carried out. The initial values of conversion and selectivity, as well as the evolution during the initial period, are detailed in Figures S4–S6 for both catalyst mixtures, and the results suggest that either temperature or the ratio between catalytic functions should be adjusted to ensure full methanol conversion at the tested conditions. The steady-state performance of both tandem systems using the same ZnO:ZrO_2_ mixed oxide is summarized in Figure 2, and details can be found in Figures S7 and S8. Please note that 80 h experiments suggested no significant changes beyond 12–20 h on stream irrespective to the GHSV used (Figure S9). At steady state, CO_2_ conversion and CO selectivity did not show any significant variation with the defined MAPO-18 space time (in g_MAPO-18_ h mol_CO_2__^–1^ at constant CO_2_/ZnO:ZrO_2_ ratio, Figure 2a) and follow the same evolution with temperature (Figure 2e), corroborating the negligible influence of the zeotypes on the reactions that take place over the oxide function. While both MAPO-18 zeotypes exhibit similar stability in CO_2_ hydrogenation, there are notable differences in hydrocarbon distribution. MgAPO-18 reduces the amount of paraffins in the effluent, favoring the propylene/propane ratio (Figure 2b) at all tested temperatures (Figure 2f). Hydrogenation of olefins over the ZnO:ZrO_2_ oxide may well be discarded as the production of paraffins increases with MAPO-18 space time (Figure S4) and the available H_2_ and oxide sites are constant in all reactions. Therefore, SAPO-18 acid sites are apparently more prone to forming paraffins through hydrogen transfer, with parallel aromatics formation (not observed due to the severe shape selectivity of the AEI framework), or direct hydrogenation over strong acid sites.

In both cases, the selective production of C_3_ hydrocarbons smoothly drops with the MAPO-18 space time, with slightly higher values for the SAPO-18 zeotype (Figure 2c). The clearly promoted formation of the C_4_ fraction for the MgAPO-18 may explain it and be attributed to (i) a higher and more selective methylation activity of the Mg version (previously observed in the AFI structure of MgAPO-5 *vs* SAPO-5);^23^ (ii) the more distorted eight-membered ring windows of the AEI structure with Mg in the lattice;^15^ or (iii) the higher strength of Mg-derived Brønsted acid sites, which could favor secondary reactions as C_2_ dimerization at low temperatures,^17^ and C_4_ cracking when the temperature was increased up to 400 °C (Figure 2g). Interestingly, iso-C_4_ were only identified when the SAPO-18 zeotype was used, and in fact, its production is favored with high SAPO-18 space time (Figure 2d) and low temperature (Figure 2h). We ascribe this phenomenon to the potential presence of Brønsted acid sites on the external surface SAPO-18 crystals as the kinetic diameter of isobutane (5.28 Å)^34^ is bigger than the eight-membered ring window of the AEI structure, even in the case of the Mg-containing distorted ring.^15^

To further study the interaction between these hydrocarbons and the zeotypes, TAP experiments were performed. Under TAP reactor conditions of high vacuum (10^–8^–10^–6^ mbar) and using a thin layer of catalyst, bimolecular and secondary reactions are minimized so that the interaction between reactants and acid sites can be characterized in the low-coverage limit.^35,36^ The pulse response for propene over MgAPO-18 is significantly slower than that observed when probing the SAPO-18 zeotype (Figure S10a), which is the consequence of the higher strength of the Brønsted acid sites originated from the isomorphic substitution of Al by Mg.^23^ The higher strength of the Brønsted acid site originating from Mg substitution was further demonstrated in our computational results where protonation of the cyclic ketene 6-fulvenone was observed in AIMD simulations performed with MgAPO-18 but not with SAPO-18 (Figure S26). Electronic energies confirmed this protonation to be favored in MgAPO-18 and disfavored in SAPO-18. Furthermore, the steric constraints of the small windows in the AEI framework allow isobutene molecules to interact only with external acid sites under TAP conditions. The residence time of isobutene on MgAPO-18 was significantly shorter than on SAPO-18 despite the lower intrinsic acid strength of the latter (Figure S10b), suggesting that the number of external acid sites accessible for isobutene is significantly higher in SAPO-18.

**Figure 3:**
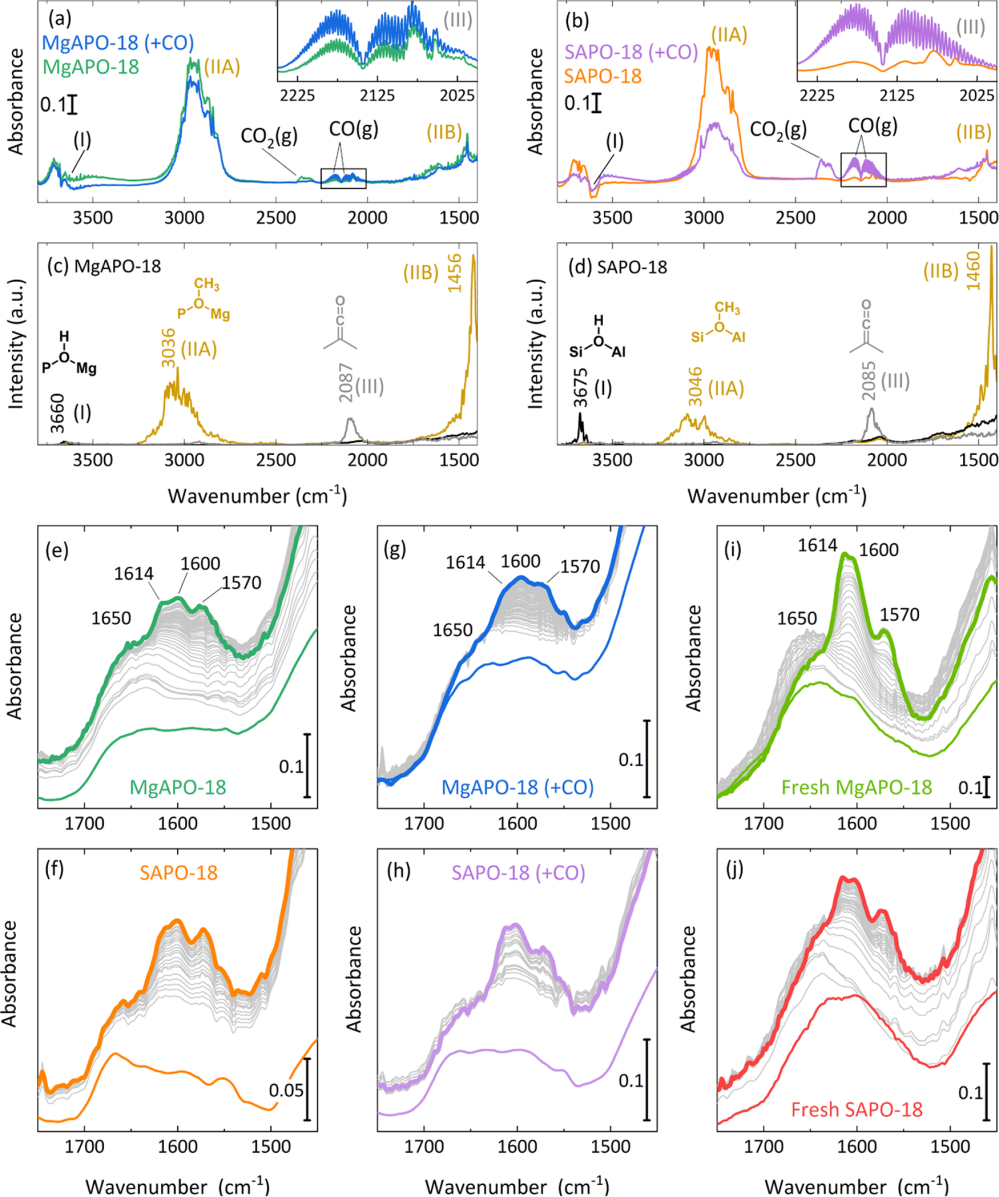
Time-resolved *in situ* IR spectra during the early stages of methanol conversion before the formation of hydrocarbons using MAPO-18 (M = Mg, Si) zeotypes: calcined and stored (a) MgAPO-18 and (b) SAPO-18 at MTO conditions and cofeeding CO. Simulated power spectra of a Brønsted acid site, surface methoxy species, and dimethylketene molecular species on the (c) MgAPO-18 and (d) SAPO-18 zeotypes. Time-resolved *in situ* IR spectra during 1 h of methanol conversion over calcined and stored (e) MgAPO-18 and (f) SAPO-18 at MTO conditions, over calcined and stored (g) MgAPO-18 and (h) SAPO-18 cofeeding CO; and *in situ* calcined (i) MgAPO-18 and (j) SAPO-18 at MTO conditions. 400 °C, 1 bar, MeOH vapor pressure at 25 °C in pure N_2_ (60 cm^3^ min^–1^) or mixed with CO (20 cm^3^ min^–1^) flow. Colored lines refers to initial (thin lines) and last (bold lines) acquisition.

Although the huge impact of methanol and water concentration explains the similar performance of both zeotypes in the tandem CO_2_ hydrogenation (Figure 1c,d), and the changes in product distribution are the consequence of the different strength and location of acid sites, the role of CO cofeeds and its potential effect on MgAPO-18 stability (Figure 1a,b) and product distribution under the MTO condition^15^ remain to be explained. The Mg- and SAPO-18 zeotypes were therefore interrogated under a series of *in situ* and *ex situ* characterization techniques. We first monitored the evolution of the MTO reaction with and without cofeeding CO by means of *in situ* FT-IR. In the early stages of the reaction (before the detection of hydrocarbon in the spectra), relevant changes were observed when cofeeding CO (Figure 3a,b). To identify and correctly assign the observed IR bands, we performed a series of AIMD simulations on relevant species to deduce the power spectra. The spectra of the pristine Brønsted acid site (BAS), surface methoxy species (SMS), and dimethylketene are plotted in Figure 3c,d (all simulated spectra, including additional species, can be found in the Supporting Information). The consumption of BAS is noteworthy in all cases (*ca.* 3660 cm^–1^, band I), as well as the formation of SMS (*ca.* 3040 cm^–1^, band IIA; and 1460 cm^–1^, band IIB). Interestingly, gas phase CO and ketene (insets and band III) are observed in the MTO reaction (without cofeeding CO). This species has been identified as the primary source of the initial C–C bond formation during the MTO,^37^ with CO being formed through decarbonylation of formaldehyde-derived compounds,^38,39^ and subsequently reacting with the already formed SMS.^39,40^ Moreover, the presence of gas phase CO was already observed when feeding methanol in isomorphically substituted MAPO materials.^18^

While no big changes were observed in the spectrum when CO was cofed with methanol over the MgAPO-18 zeotype (Figure 3a), the ketene band is no longer present in the case of the SAPO-18 zeotype (Figure 3b), and gas phase CO_2_ surprisingly increases. Liu et al.^41^ attributed the presence of CO_2_ to decarboxylation pathways also originating from the reaction of formaldehyde and surface acetate species (product of methoxy carbonylation) *via* acrylic acid. Recently, Huber and Plessow^39^ computed similar barriers for carbonylation and carboxylation pathways over SSZ-13, although they proposed carboxylation to be more likely *via* the propiolactone intermediate. Our results suggest that in the case of SAPO-18, the presence of CO increases the rate of methoxy conversion to ketene, which is rapidly converted—presumably with formaldehyde—leading to olefin formation by the preferred decarboxylation route (versus decarbonylation). A higher involvement of formaldehyde-mediated reactions may also be the reason for the promotion of the Prins reaction, the bigger role of the aromatic cycle (see the lower C_4_/C_2_ ratio of SAPO-18 at a low temperature in Figure 2g), and therefore the faster deactivation (Figure 1a,b), all in agreement with the previous results from the Lercher group.^41^ Otherwise, the lack of CO_2_ over MgAPO-18 suggests that the CO-mediated carbonylation/decarbonylation reaction cycles^3^ should be the main source of the first olefins and that decarboxylation pathways are less favored in this case. This, together with our previously observed faster methylation rates on Mg-substituted AlPO materials,^23^ may explain the longer lifetime of MgAPO-18.^41^ Furthermore, the ability to scavenge formaldehyde *via* CO/H_2_ formation has been proposed by Hwang and Bhan^42^ as a strategy to avoid triggering formaldehyde-mediated deactivation routes. Therefore, a preferred formaldehyde degradation to CO/H_2_ over the potential Mg(II)-based Brønsted/Lewis acid–base site pair^15^ may also explain the lack of CO_2_ formation and the higher performance stability of the MgAPO-18 zeotype.

**Figure 4:**
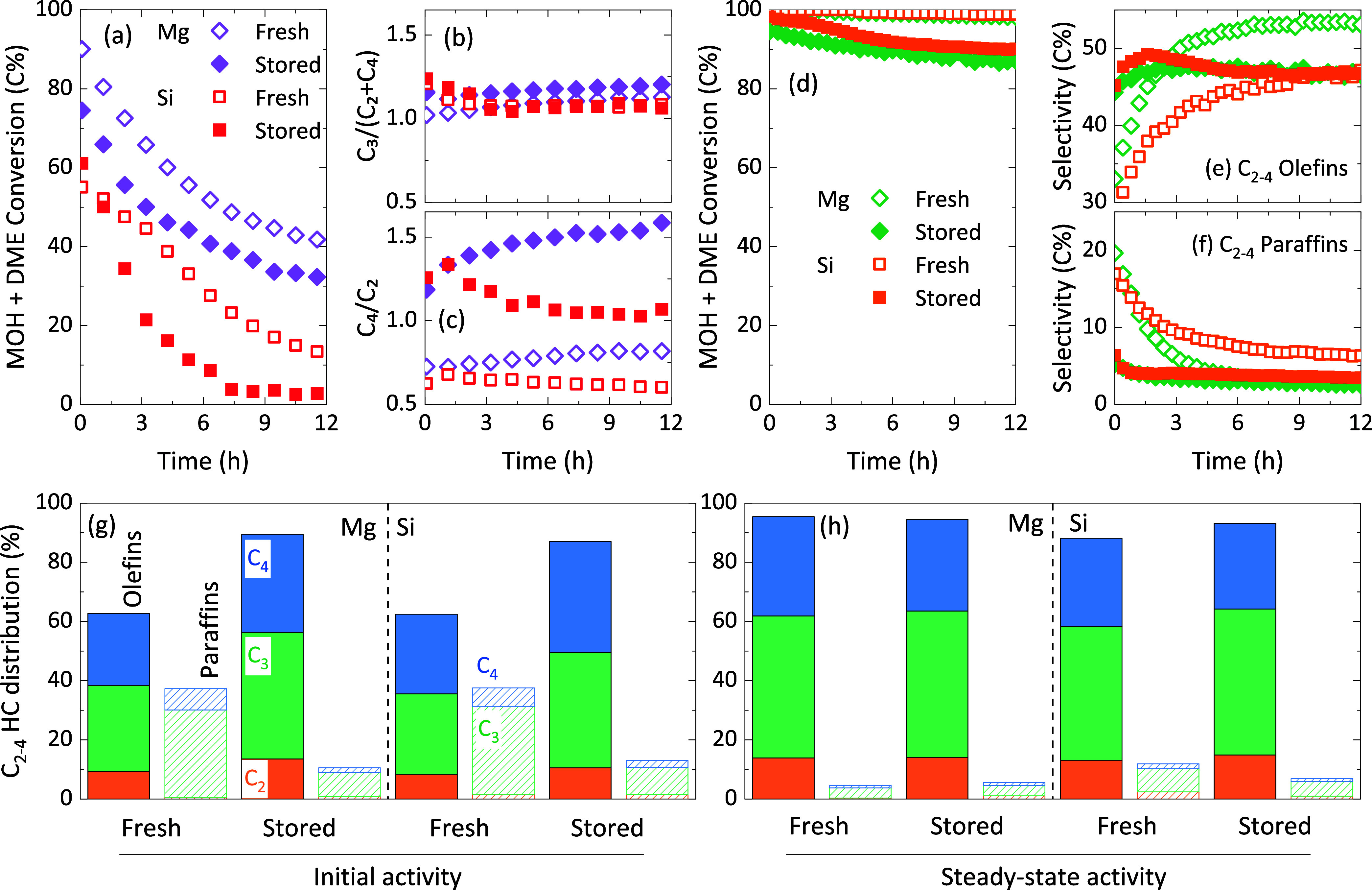
Comparison of the freshly calcined and stored MAPO-18 (M = Mg, Si) zeotypes during the methanol-to-olefins and the tandem CO_2_-to-hydrocarbon reactions. Evolution with time of the (a) oxygenate conversion and (b) C_3_/(C_2_ + C_4_) and (c) C_4_/C_2_ ratios during the methanol-to-hydrocarbons reaction (350 °C, 1 bar, 40 mbar MeOH in He, 6.5 g_MeOH_ h^–1^ g^–1^). Evolution with time of the (d) oxygenate intermediate conversion, (e) C_2–4_ olefins and (f) C_2–4_ paraffins, and (g) initial and (h) steady-state C_2–4_ olefin and paraffin distribution during the tandem CO_2_-to-hydrocarbon reactions using ZnO:ZrO_2_ + MAPO-18 catalysts in a 1/1 catalyst ratio (350 °C, 30 bar, 12000 cm^3^ h^–1^ g^–1^, H_2_/CO_2_ ratio of 3). CO_2_ conversion of 5.0 ± 1.0% in all reactions for the sake of comparison.

The evolution of hydrocarbon species formation was also monitored during 1 h of reaction, and the IR spectral region of C=C stretching modes between 1700 and 1400 cm^–1^ is depicted in Figure 3e–j. The main components of HCP species that quickly evolve in the first stages of the conversion can be monitored, as already discussed in a previous study.^18^ Four main bands were assigned to characterize the reaction: 1650 cm^–1^ corresponding to the C=C stretching of long-chain olefins^43^ and 1614–1600 and 1570 cm^–1^ corresponding to collective ring C=C vibrations. Under pure methanol/N_2_ feed, both MgAPO-18 (Figure 4e) and SAPO-18 (Figure 4f) show a similar performance, with the olefin C=C stretching band (1650 cm^–1^) growing first, followed by the appearance of aromatic bands (1614–1600 and 1570 cm^–1^). The olefin band remains constant while aromatic species are formed. Experiments carried out under ^13^C-labeled methanol and similar conditions allowed us to confirm the same performance of the two zeotypes by solid-state ^13^C NMR (Figure S11). For MgAPO-18 and SAPO-18, the NMR spectra were almost superimposable, suggesting the formed hydrocarbon pool species in the zeotypes are identical in the absence of CO.^44−47^ Moreover, the presence of aromatics and alkyl groups was confirmed after 30 min on stream, as one would expect from the methanol-to-hydrocarbons chemistry.

Again, the evolution of the hydrocarbon species changed when experiments were conducted by cofeeding CO (Figure 3g,h, for MgAPO-18 and SAPO-18, respectively). This time, very slow spectral changes are observed, and the 1650 cm^–1^ band (olefin C=C stretching) is totally missing. Very slowly, the aromatic components are directly formed, without visible formation of long olefins as observed before. At the end of the experiment, only the components at 1614–1600 and 1570 cm^–1^ are present with comparable (quite low) intensity. A direct action of CO in the mechanism of MTO is therefore suggested not only for the initiation of the C–C bond formation but also for hydrocarbon chain growth. Our results suggest that oxygenate intermediates prevail over alkenes during the chain reaction mechanism,^18^ and the presence of CO favors the evolution of aromatic compounds versus the olefin-mediated route.^48^

### Stability of the Heteroatoms in the MAPO-18 Zeotypes

In a previous work featuring a CoAPO-18 zeotype,^18^ we learnt the high tendency of the Co(II) species in the AlPO-18 framework to coordinate with water and how exposing the template-free material to air and then removing the water irreversibly affect the BAS/LAS concentration. Therefore, to analyze this effect in the MgAPO-18 and SAPO-18 versions, and the potential implications on catalytic activity, we extended our study to zeotypes calcined and stored differently. First, we carried out *in situ* MTO reactions followed by IR along with performing an *in situ* calcination. That is, pellets of the catalysts were prepared with the as-made MAPO-18 zeotypes, calcination with air was carried out following the same conditions we used before, and without exposing the samples to air, reactions were started. Unfortunately, due to setup limitations, *in situ* calcination and CO cofeed were not allowed. The results for MgAPO-18 and SAPO-18 are, respectively, shown in Figure 3i,j. Similar to what was observed for the CoAlPO-18 zeotype,^18^ the conversion in the presence of the *in situ* calcined MgAPO-18 is very quick and produces intense signals in the hydrocarbon regions. Here, a broad olefin signal growing (1650 cm^–1^) is first observed, which is well-defined and intense for MgAPO-18 and lower for SAPO-18. During the progress of the reaction, the signal at 1650 cm^–1^ is gradually substituted by the bands growing at 1614–1600 and 1570 cm^–1^ (collective ring C=C vibrations), which become extremely intense in the case of MgAPO-18. This indicates the condensation of the olefins to form aromatic cycles; indeed an isosbestic point is clearly visible for MgAPO-18 when the olefin signal disappears, and aromatic bands start growing. In the case of SAPO-18, the olefin signal remains constant when the aromatic formation starts, so we do not identify an isosbestic point, and this indicates the contemporary presence of olefins and aromatics all over the remaining reaction time. At the end of the experiment, the two samples present a signal at 1570 cm^–1^ with comparable intensity, while the components at 1614–1600 cm^–1^ are much more intense in MgAPO-18 (almost double the intensity of SAPO-18). The formation of aromatics (main hydrocarbon pool species) is therefore faster than that observed with the calcined and stored samples (Figure 3e,f).

From these results, it seems clear that, especially in the MgAPO-18 case, the catalyst activity toward the formation of the hydrocarbon pool seems to decrease upon storage of the calcined samples. At flow reaction, we noticed certain differences in the catalytic performance only after storing samples for longer periods of time (>3 months). At MTO conditions, both catalysts show slightly longer lifetimes when they are freshly calcined (Figure 4a, detailed product distribution in Figure S12), and while the selectivity toward propylene with respect to ethylene and butenes was maintained constant (Figure 4b), the C_4_/C_2_ ratio clearly decreases (Figure 4c). Ethylene is usually attributed to dealkylation of heavier polymethylbenzenes or cracking of longer hydrocarbons,^16,41,49^ both requiring sites of strong Brønsted acidity, therefore suggesting a loss of this type of sites upon storage.

The effect of storing the catalyst is clearer when the materials were tested along with the ZnO:ZrO_2_ mixed oxide in the tandem CO_2_ hydrogenation to hydrocarbons due to the wider hydrocarbon distribution (with olefins and paraffins; Figures S13–S14). During the first 12 h on stream, oxygenate intermediate conversion drops in both cases (Figure 4d), yet far from the deactivation rates and differences between the zeotypes observed at MTO conditions. As previously discussed, the lower methanol partial pressure and presence of water equalize the performance of Mg and Si versions of the zeotype, but the high partial pressure of H_2_ influences the hydrocarbon distribution in both cases. Two main differences must be highlighted between stored and freshly calcined samples: (i) higher amounts of unconverted methanol are identified in the product effluent with the stored samples (Figure 4d); and (ii) a more pronounced and longer unsteady period (after hydrocarbon pool development), in which the product distribution evolves until steady conditions are reached, is observed for the freshly calcined samples, with significant production of saturated paraffins initially (Figure 4e,f). Both features suggest the presence of more and/or stronger acid sites in the freshly calcined samples as we previously discarded the idea that this hydrogenation may originate in the ZnO:ZrO_2_ sites (Figure S4). Taking a closer look at the hydrocarbon distribution (Figure 4g), C_3_ hydrocarbons are the main fraction of both olefinic and paraffinic products. Interestingly, the product distribution evolves differently for the freshly calcined and stored samples. A faster deactivation of the secondary reactions of paraffin formation is observed for the freshly calcined samples (Figure 4f), which results in almost the same hydrocarbon distribution for freshly calcined and stored samples after the steady state is reached (Figure 4h), the relevant conditions to compare catalysts and those used for the discussion of heteroatoms’ role above. Material degradation was observed for both materials, yet it was particularly faster for the Mg version of the zeotype (months *vs* 1 year range). Therefore, the observed formation of paraffins now with the stronger sites of MgAPO-18, the suggested absence of external sites, and its faster degradation suggest that these stronger sites are the most prone to degradation and are somewhat labile. Nonetheless, despite the partial loss upon storage, the steady-state performance of the catalyst is barely affected, which we attribute to the more stable acid sites. The higher affinity of the harder Mg^2+^ cations for water is suggested as a potential cause for this faster degradation (even the humidity in the atmosphere). For this reason, and in view of these results (Figures 3 and 4), a more controlled characterization of the materials was performed.

**Figure 5:**
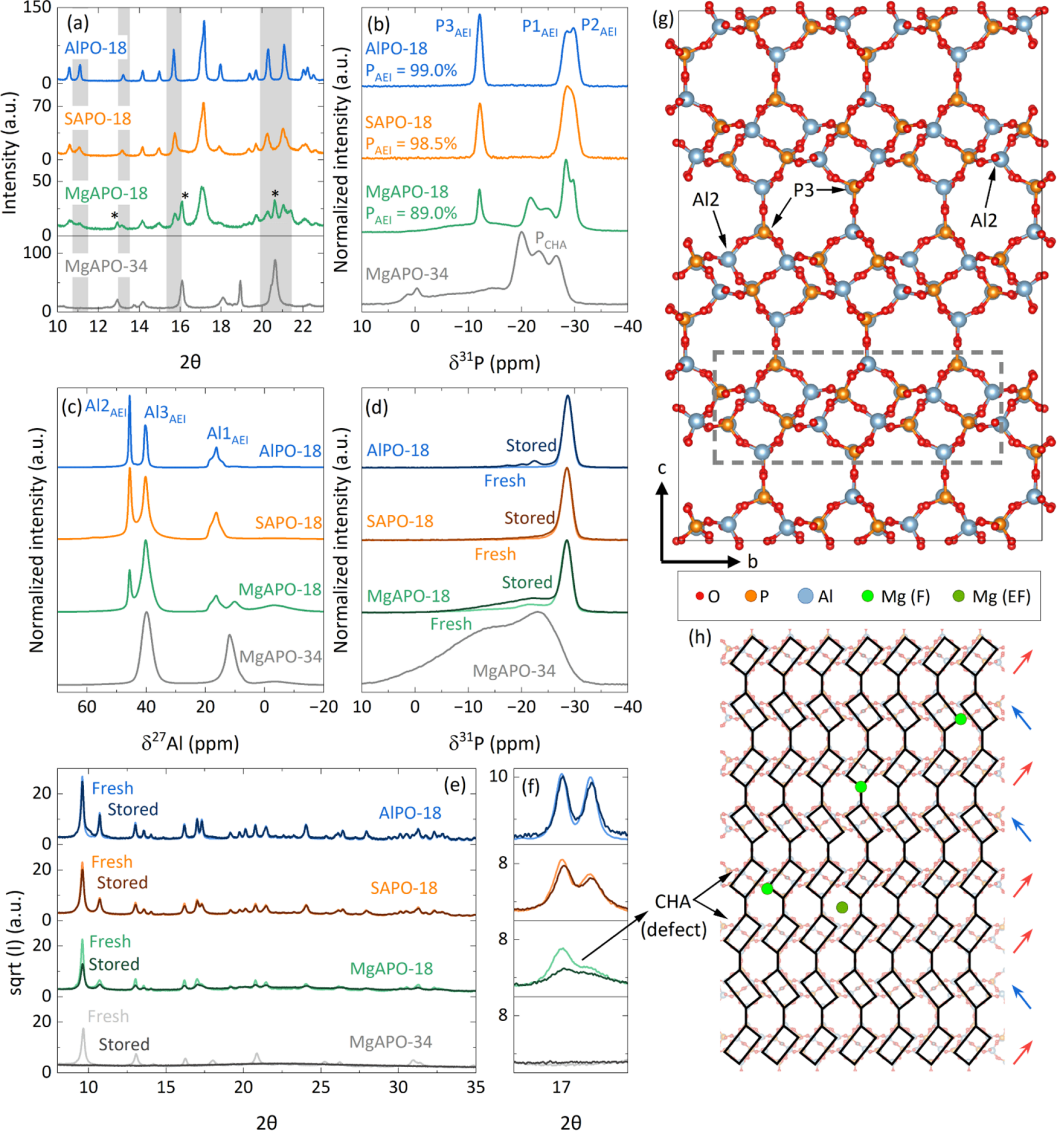
(a) XRD diffraction patterns, (b) ^31^P NMR, and (c) ^27^Al-NMR of the as-made AlPO-18 (blue), SAPO-18 (orange), MgAPO-18 (green), and MgAPO-34 (gray). Comparison between freshly calcined and stored zeotypes by (d) ^31^P NMR and (e) XRD diffraction. (f) Details of the XRD diffraction pattern. (g) Model of the AEI structure and (h) sketch of the AEI stacking model, including CHA defects. Rectangles illustrate the double six-membered rings of the AEI structure along the *a*-axis, and green spheres represent potential Mg locations in the framework (F) and extra-framework (EF).

First, the characterization of the as-made samples (before calcination, with the structure-directing agent) by XRD already showed differences between the samples. The neutral AlPO-18, synthesized as a reference material, is a pure phase material with an AEI topology, confirmed by Rietveld refinements (Figure S15 and Table S2; atomic model for refinement extracted from Simmen et al.^50^). The as-made SAPO-18 diffraction pattern also agrees with the pure AEI phase; however, the slightly broader and less well-defined features (see shadowed reflections among others in Figure 5a and the full diffraction pattern in Figure S15) suggest smaller crystallites and perhaps some minor CHA defects.^51^ The general features of AEI became even broader in the case of the as-made MgAPO-18. Not only that, new reflections appeared (2θ values of 16, 20.8, and 21.7). The M doping is well-known to increase the degree of stacking probability in this family of materials,^51,52^ and the presence of AEI/CHA intergrowth architectures was previously described with high-resolution STEM by Shen et al.^53^ The hypothesis of CHA defects in the samples, mainly in the Mg version, motivated us to synthesize a pure MgAPO-34 sample. The comparison of the XRD patterns (Figure 5a) now confirms that the new reflections can be attributed to a CHA topology, although the absence of some CHA reflections in the MgAPO-18 diffractogram (see the region between 18 and 20 2θ) implies that the material is not composed of a simple mixture of two crystalline phases.

Solid-state NMR, which probes the local environment of the structure, corroborated the presence of CHA defects in the MgAPO-18 zeotype. The three unique signals in the ^31^P NMR spectra, observed for AlPO-18 and SAPO-18 (Figure 5b), correspond to the typical phosphorus local environments of pure AEI (almost 99% quantified), which imply a monoclinic distortion (assignment of peaks based on He and Klinowski^54^). New extra P signals with chemical shifts in the −20 to −26 ppm range appeared in the ^31^P NMR spectrum of the MgAPO-18 zeotype. Indeed, only 89% of the P presented bond lengths and angles suitable within the AEI framework. A comparison of the ^31^P NMR spectrum to that of the MgAPO-34 zeotype suggests that the new signals belong to the CHA topology. A fourth Al species is present in the ^27^Al-NMR spectrum of MgAPO-18, also matching with the CHA phase (Figure 5c). Accordingly, the distortions at both the global (XRD) and the local (NMR) level increased with the presence of Mg in comparison to Si.

It might also be argued that P3 and P2 positions are more affected by the heteroatom substitution since in both cases the relative intensities of the signals at −10 and 30 ppm decreased (Figure 5b). Si would naturally substitute phosphorus, creating BAS. Mg would otherwise exchange aluminum, and only the Al2 signal, at −45 ppm, was progressively reduced (Figure 5c). However, the possibility of Al3 substitution cannot be discarded since the emerging CHA defects mask that region in the ^27^Al-NMR spectrum (Figure 5g). Note that Al1 and P3 are “equivalent” positions connecting two double six-membered rings through an O bond, constituting the neighboring layers of the typical AEI and/or CHA stacking sequences (detailed gray square in Figure 5g).

Right after calcination (freshly calcined samples), the differences between the materials became more subtle. ^31^P NMR results suggest a relaxation of the β angle for the three materials, also supported by Rietveld refinements (Figure S16 and Table S2). The spectra present a strong signal at *ca.* −28.6 ppm (Figure 5d). Interestingly, the two extra weak signals at −21.3 and −13 ppm in the MgAPO-18 could also suggest smaller distortions at the local level in the material compared to the as-made sample. By XRD (Figure 5e), all reflections could be fitted with the AEI orthorhombic empty framework. However, some reflections as the [111] (at 2θ 10.5°) and [113] (detailed in Figure 5f) show misfit because of their abnormally large full width at half-maximum (fwhm), especially in the case of MgAPO-18 (Figure S16). These features have been attributed to the presence of CHA defects in the framework (0.05 probability approximately).^55^ The storage of SAPO-18, MgAPO-18, and MgAPO-34 zeotypes after calcination led to significant local distortion (Figure S17) and apparent loss of their crystallinity (Figure S18, note the raw intensity differences). This phenomenon is well-known for AlPO-18 materials and is attributed to a rehydration of the framework upon water exposure, which causes a lack of long-range order due to water disrupting the framework.^56^ SAPO-18 and MgAPO-18 recovered from the distortion at the local level (see NMR data in Figures 5d and S19), and the long-range order is restored upon drying the stored materials (stored in Figure 5e). The key fwhm indicating CHA defect presence became less prominent upon drying the stored MgAPO-18 zeotypes. Therefore, a fading of the CHA defects is suggested when drying the stored MgAPO-18 (Figure 5f). The same effect was not as clear in the SAPO-18 zeotype.

In contrast to the long-range order recovery observed in AEI materials upon water removal from the stored samples (Figure S19), MgAPO-34 collapsed to a fully amorphous material (black line in Figure 5e). Because MgAPO-18 contains more CHA defects than SAPO-18 and these units seem to collapse more easily after drying, it comes as no surprise that the stacking probability also decreases after drying. Note that the overall crystallinity is not totally recovered after exposing the samples to water and drying them (decrease in XRD intensity from freshly calcined to stored and dried samples). All of this suggests that these defects are weak points that potentially break the crystallites upon water exposure (Figure 5h). This could explain the evolution of the zeotype properties upon storage, particularly fast in the case of MgAPO-18, with the highest CHA defect concentration.

Due to the relevance of the presence of water in both methanol-to-olefins and tandem CO_2_-to-hydrocarbons processes, and the speculation of zeotypes being degraded in the presence of water, we performed reaction–regeneration cycles for both processes, confirming that the materials preserved their integrity during reaction cycles. In fact, the OXZEO catalysts recover the initial activity when using both MgAPO-18 and MgAPO-34 zeotypes (Figure S20), and the MTO activity was preserved after regeneration in air at 550 °C and zeotype steaming at 350 °C (Figure S21).

**Figure 6:**
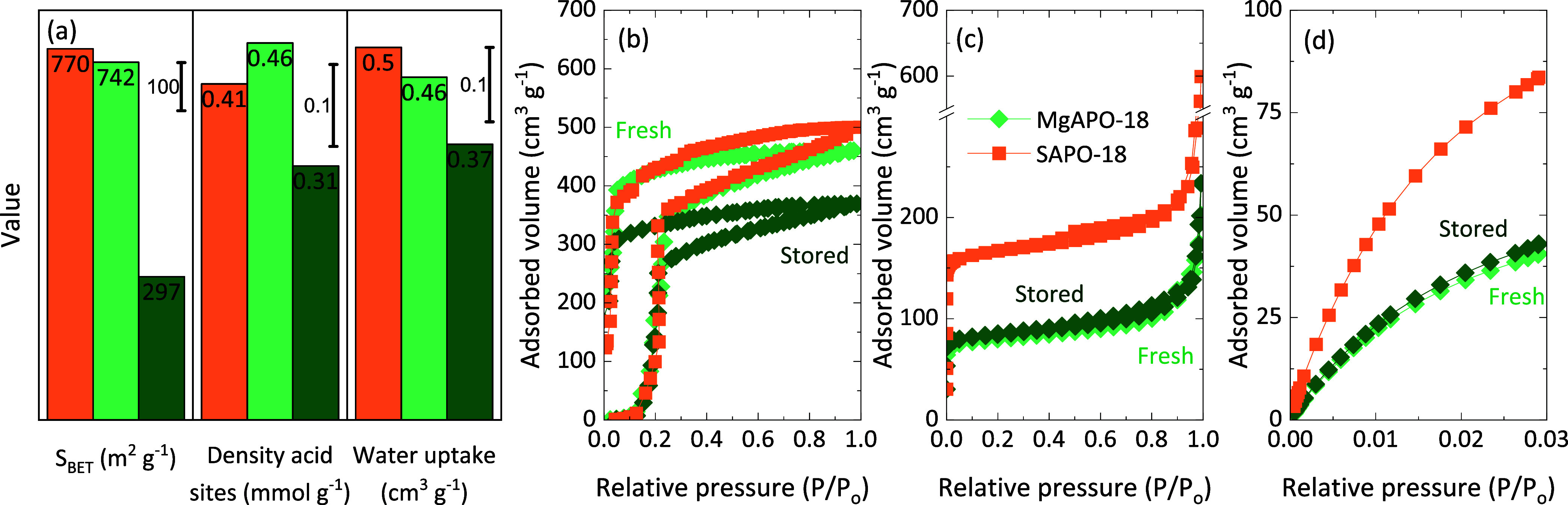
(a) BET specific surface area, density of acid sites, and water uptake of freshly calcined MgAPO-18 and SAPO-18 and stored MgAPO-18. (b) Water adsorption–desorption isotherms at 25 °C and (c) N_2_ adsorption–desorption and (d) CO_2_ adsorption isotherms of the samples after water adsorption–desorption experiments.

To further understand how water interacts and degrades these materials upon storage, adsorption–desorption experiments of different adsorbates were carried out. Freshly calcined and stored MgAPO-18 were analyzed, and a freshly calcined SAPO-18 was also characterized for the sake of comparison. Both freshly calcined samples have similar BET specific surface areas with 770 and 742 m^2^ g^–1^ (Figure 6a). The collapse of the structure is evident for the stored MgAPO-18 zeotype (297 m^2^ g^–1^) and in agreement with the loss of crystalline domains (Figure 5e). In a similar fashion, the density of Brønsted acid sites by propylamine titration decreases upon storage, already suggesting a certain loss of lattice Mg. Here, we must mention that according to the nominal heteroatom loading, the density of Brønsted acid sites should have been *ca.* 0.62 mmol g^–1^; therefore, the presence of extra-framework Si and Mg cannot be discarded. Water adsorption–desorption isotherms at room temperature (25 °C) are shown in Figure 6b. Similar isotherm shapes are exhibited by the three materials, with the main adsorption range being located at 0.2 relative pressure. Clearly, the stored sample adsorbs the lowest amount of water in this range, related to the loss of micropores. The total water uptake decreases from 0.46 to 0.37 cm^3^ g^–1^ upon storage (Figure 6a). The slightly lower water adsorbed by the Mg version is mainly related to the mesoporous range and can be ascribed to an incipient loss of mesoporosity by this sample. All materials showed a similar hysteresis loop for water desorption, suggesting the high affinity of these materials to water.

The structural and textural properties of the samples were investigated after the water adsorption–desorption experiments. As an example, Figure S22 shows the diffractograms of the MgAPO-18 during recalcination of the samples collected by *in situ* XRD: (i) right after the water adsorption–desorption experiments; (ii) at 500 °C (maximum temperature of recalcination); and (iii) at 25 °C after the heat treatment. Initially, the MgAPO-18 zeotype shows a similar distortion due to the presence of water to that observed in Figure S18 upon storage. Again, long-range order is recovered after calcination. The presence of water within the pores is also suggested by the isotherms in Figure 6b. Nevertheless, the most interesting feature of the materials was also confirmed by the characterization of the porous texture. The freshly calcined and stored MgAPO-18 show almost overlapped N_2_ adsorption–desorption (Figure 6c) and CO_2_ adsorption isotherms (Figure 6d), indicating that their final porous and narrow micorporous structure is very similar. Indeed, the estimated textural parameters are almost the same (Table S4) and, most importantly, very close to the initial stored MgAPO-18 (Table S3), with *ca.* 300 m^2^ g^–1^ BET surface area. This means that the stored zeotype barely changes, while the freshly calcined one degrades to the same final state. The low stability of the presumably most active Mg-derived Brønsted acid sites is reinforced. However, these sites are mainly responsible for undesirable secondary reactions during the tandem CO_2_-to-hydrocarbons process that can be minimized while the olefin-making sites are still stable upon storage.

The presence of Brønsted acid sites makes it obvious that most Mg is originally substituting Al in the framework. However, there is not enough evidence to pinpoint the structural details of Mg location. Our hypothesis is that the extra-framework Mg may exist as Mg(OH)_6_^2+^, stabilizing CHA defects in the case of calcined samples exposed to the atmosphere. After CHA collapse, these cations may be deposited in the imperfect external surface of the crystallite. However, most of the Mg should be fixed within the framework, preferentially in the bulk of the crystals rather than in the external surface. The lack of a stable external lattice Mg is consistent with the absence of iso-butanes/butenes during the reaction (Figure 2). The hard character of Mg^2+^ cations, with a strong preference toward OH^–^ or H_2_O over O^2–^ in solution,^57^ may lead strong Brønsted acid sites −Mg–O(H)–P– to form stable site coordination in the form of −(HO)Mg–O–P(OH)– at both reaction and storage conditions. Our results suggest that the active Brønsted acid sites can be replenished in reaction–regeneration cycles, but they are however torn (by water) after certain time upon storage, to which the existence of stacking faults may contribute. This may well be the result of the extremely hydrophilic metastable framework Mg that can form some sort of hexacoordinated complex when the long-range order of the structure is lost upon hydration and can be partially restored after drying, when the global structure is recovered.

## Conclusions

In this contribution, a family of MAPO-18 (M = Mg, Si) zeotypes was studied for methanol conversion to olefins under MTO, methanol/CO/H_2_ cofeed, and CO_2_ hydrogenation conditions, separately, the latter in tandem with a ZnO:ZrO_2_ catalyst. Powder XRD and solid-state NMR showed that the as-prepared SAPO-18 consists of a pure-phase AEI topology, possibly containing minor CHA defects. Si^4+^ occupies mainly P2 and P3 positions in the AEI framework. Otherwise, the as-prepared MgAPO-18 consists of crystallites of faulty AEI topology, containing more CHA defects, which were confirmed by the synthesis and characterization of a pure MgAPO-34 material with a CHA structure. Mg^2+^ occupies the Al2 position in the AEI framework and may potentially occupy the Al3 position (undisclosed due to peak overlap). After long-term storage, distortion at the local level and loss of the long-range order were observed for both SAPO-18 and MgAPO-18. While distortion is mostly recovered for SAPO-18 after subsequent drying, MgAPO-18 showed signs of faster degradation upon storage. Combining characterization studies of MgAPO-18 with parallel studies of MgAPO-34 suggests that the CHA domains in MgAPO-18 collapse during the storage–drying cycle. It is of interest to note that very similar material changes were observed after the long-term storage of MgAPO-18, followed by drying, and after exposing freshly calcined MgAPO-18 to water sorption experiments at 25 °C, followed by drying, confirming that CHA defects could contribute to the faster degradation of the MgAPO-18 zeotype compared to its Si counterpart.

Considering next the catalytic performance of the two materials, testing under MTO conditions led to 50 and 100% loss of initial activity after 12 h on stream for MgAPO-18 and SAPO-18, respectively. Addition of high-pressure CO/H_2_ to the methanol feed substantially stabilized the performance of MgAPO-18 with only 10% activity loss, while SAPO-18 still lost 90% activity after 12 h on stream. In a previous study, it was demonstrated that H_2_ mitigated coke formation over MgAPO-18, while CO addition mitigated olefin hydrogenation over both catalysts. Conversely, testing under CO_2_ hydrogenation conditions, in tandem with a ZnO:ZrO_2_ catalyst, yielded a similar performance of the two catalysts, which was ascribed to two effects: first was the coke-mitigating role of high H_2_O/CH_3_OH ratios throughout the catalyst bed, which stabilized the performance of SAPO-18, and second was the deactivation of strong BAS, especially from MgAPO-18. A comparison of the effluent composition from both catalysts under CO_2_ hydrogenation conditions showed that the freshly calcined materials were more active for methanol conversion, as well as for olefin hydrogenation reactions. The hydrogenation activity was gradually lost and stabilized at <5% selectivity after 12 h on stream. Notably, the steady-state selectivity of freshly calcined and stored materials converged to very similar levels. After the initial deactivation of BAS, stable performance was observed before/after exposure to steaming/regeneration cycles.

Detailed mechanistic studies were carried out to unravel differences between the materials under various reaction conditions. Under MTO conditions, solid-state ^13^C NMR and *in situ* IR spectroscopy studies strongly indicated a similar initial buildup of the HC pool species in the two catalysts: olefins were formed first, followed by a gradual formation of aromatic species, to a mixture of aliphatic and aromatic species during the first hour on stream. When adding CO to the methanol feed at atmospheric pressure, *in situ* IR studies suggested that olefin formation was suppressed over both catalysts and replaced by aromatic formation even in the first hour of the reaction. On the other hand, a*b initio* molecular dynamics simulations combined with IR spectra revealed important mechanistic differences between the two catalysts, in particular under CO cofeed conditions. Both catalysts formed CO and ketene when methanol was fed alone, but in the presence of cofed CO, the SAPO-18 catalyst seemed to promote a decarboxylation mechanism, typical of formaldehyde-based chain growth, while the MgAPO-18 catalyst seemed to promote a decarbonylation mechanism, typical of methanol/ketene-based chain growth.

## Experimental Section

### Catalyst Synthesis

The ZnO:ZrO_2_ mixed oxide was synthesized by a coprecipitation method. An aqueous mixture of ZrCl_4_ and Zn(NO_3_)_2_ with a Zn/(Zn + Zr) ratio of 10 at% was added dropwise to a 5 M NaOH solution with 50% base excess. The solution was stirred at 100 °C for 48 h, after which a white precipitate was observed in the slurry. The powder was filtered and washed 3 times in a 0.2 M NH_4_NO_3_ aqueous solution. Finally, the washed powder was dried at 110 °C overnight and calcined isothermally at 500 °C for 6 h using a temperature ramp of 1.5 °C min^–1^.

MAPO-18 and neutral AlPO-18 (AEI structure) were prepared using the hydrothermal synthesis protocol presented in our previous work.^15^ Briefly, phosphoric acid (85 wt %, Merck), deionized water, and N,N-diisopropylethylamine as a structure-directing agent (>99 wt %, Merck) were first mixed. Pural (AlO(OH), Sasol) was slowly added under stirring for 5 min. To obtain the neutral AlPO-18, the mixture was directly transferred to a Teflon-lined stainless-steel autoclave and heated at 160 °C under rotation for 8 days. To obtain the SAPO-18, Ludox was added to the mixture targeting a Si/(Al + P) ratio of 0.04. After 20 min of continuous stirring, the final mixture was transferred to the autoclave and heated at 190 °C for 12 h. In addition, to obtain the MgAPO-18, an aqueous mixture of Mg acetate (>98%, Merck) was prepared and added to the gel targeting the same Mg/(Al + P) ratio of 0.04. After 20 min of continuous stirring, the final mixture was transferred to the autoclave and heated at 160 °C for 8 days.

The MgAPO-34 (CHA structure) was also prepared following a hydrothermal synthesis protocol, this time using an initial solution of deionized water with the tetraethylammonium hydroxide structure-directing agent (35 wt %, Merck), aluminum isopropoxide (>98%, Merck), and phosphoric acid. After 2 h of stirring to hydrolyze the Al source, the mixture of Mg acetate was slowly added. A Mg/(Al + P) ratio of 0.04 was also targeted for this zeotype. After 20 min of continuous stirring, the final mixture was transferred to the autoclave and heated at 170 °C for 3 days. All obtained zeotypes were washed with deionized water and centrifuged three times. Subsequently, they were calcined isothermally at 550 °C for 6 h using a temperature ramp of 1.5 °C min^–1^.

### Catalytic Testing

Tandem CO_2_ hydrogenation reactions were carried out in a high-pressure test reactor (PID Eng & Tech). A mixture of ZnO:ZrO_2_ with a MAPO-18 zeotype was crushed together, in a known composition from 3:1 to 1:3, and sieved to a particle size of 250–425 μm. The mixture was then loaded in a silica-lined packed-bed stainless-steel reactor (inner diameter 9 mm), which was heated by a cylindrical ceramic oven. The temperature was measured in the middle of the catalyst bed by a type-K thermocouple. The setup was provided with a downstream pressure regulator capable of maintaining the system up to 40 bar. The temperature in the hot box was maintained at 130 °C, to preheat the gas mixtures. Prior to the catalytic testing, the samples were pretreated at 400 °C under a 10% H_2_ (in Ar) flow, after which the temperature was decreased to the reaction temperature and the pressure was increased to 30 bar. Experiments were performed at the following conditions: 325–400 °C, GSHV values of 2000–24,000 cm^3^ h^–1^ g^–1^, and zeolite space time of 1.4–12 g_MAPO-18_ h g_CO_2__^–1^. The evolution of reactions with time was monitored by a gas chromatograph (Agilent 8890 GC) connected in line with the reactor. The GC was provided with three columns (CP-Sil 8 CB, GS-GasPro, CP Molesieve 5A) and detectors (two FIDs and a TCD) and a PloyARC microreactor for the reliable quantification of CO_2_ and CO.

High-pressure cofeed experiments were carried out in a similar reactor setup (PID Eng & Tech), this time provided with a CORI-FLOW controller (Bronkhorst) to feed liquid methanol. In a typical reaction, 250 mg of MAPO-18 was crushed and sieved to the same particle size (250–425 μm), loaded into the reactor, and pretreated at 400 °C in air for 1 h. Then, the reaction runs were carried out at the following conditions: 350 °C, 30 bar (1 bar methanol in a 3/1 H_2_/CO flow), 2.5 g_MeOH_ h^–1^ g^–1^. The reaction products were analyzed in a Scion 456-GC, provided with six columns (MolSieve 13X, HayeSep Q, HayeSep N, Rt-Stabilwax, Rt-Alumina/MAPD, and Rtx-1) and three detectors (two FIDs and a TCD).

Methanol-to-hydrocarbons reactions at ambient pressure were carried out in a homemade 8 mm fixed-bed reactor setup. In a typical experiment, *ca.* 50 mg of the calcined or as-made MAPO-18 was crushed and sieved to the same particle fraction (250–425 μm) and loaded into the quartz reactor. Temperature was controlled by a type-k thermocouple located inside a quartz sleeve in the middle of the catalyst bed. The MAPO-18 zeotypes were heat-treated at 550 °C for 1 h (or calcined *in situ* following the pretreatment explained above). The reactions were then performed under the following conditions: 350 °C, 1 bar (40 mbar methanol in He), and 6.5 g_MeOH_ h^–1^ g^–1^. The evolution with time of products was monitored by a GC-MS instrument (Agilent 7890, with a FID, and Agilent 5975C, with an MS detector) connected in line and provided with two Restek Rtx-DHA-150 columns, and hydrogen (Praxair, purity 6.0) was used as the carrier gas.

Experiments of methanol conversion were monitored by *in situ* FT-IR spectroscopy in a Bruker Invenio R spectrophotometer with an MCT cryodetector working at 40 kHz and equipped with an AABSPEC 2000 multimode FT-IR cell operating in the transmission mode. The cell was connected to a mass flow controller for the direct gas dosage within the measurement chamber and allowed high-temperature *in situ* measurements. The samples were placed inside the cell in the form of a self-supporting pellet of about 10 mg of pressed powder. Both SAPO-18 and MgAPO-18 were pretreated in pure N_2_, raising the temperature from 30 to 550 °C at 5 °C min^–1^. In the case of the *in situ* calcined condition, the pretreatment was conducted in the same way, but fluxing a mixture of N_2_ (60 mL min^–1^) and O_2_ (20 mL min^–1^). N_2_ (60 cm^3^ min^–1^) or mixed N_2_/CO (20 cm^3^ min^–1^) (in the cofeeding experiments) were saturated with MeOH at 25 °C and fed directly into the AABSPEC cell where the catalysts were kept at 400 °C and ambient pressure. The reaction was followed for 1 h by continuous acquisitions of IR spectra, accumulating 32 scans for each spectrum with a 2 cm^–1^ resolution. Considering the interferometer speed of 40 kHz, each acquisition required 16 s, which was considered the time resolution for the interpretation of the spectral changes described in the Results section.

### Catalyst Characterization

X-ray diffraction (XRD) patterns were recorded using a Bruker D8-A25 in transmission capillary geometry with a Ge(111) Johanssen monochromator and a Lynxeye detector with Cu Kα_1_ radiation (λ = 1.5406). The samples were loaded in 0.7 mm borosilicate glass capillaries and collected at 25 °C equilibrated with ambient moisture. The measurements of freshly calcined zeotypes were performed *in situ* by loading the as-made material and calcining the material in an open capillary. Following the same calcination protocol, the temperature was raised up to 550 °C at 1.5 °C min^–1^ ramp in air and then maintained isothermal for 6 h. The calcined samples were flame-sealed on cooling at 200 °C inside the capillaries. Stored samples were dried at 115 °C for 12 h and flame-sealed.

N_2_ adsorption–desorption physisorption was carried out in Belsorp Mini II equipment at −196 °C. Prior to the experiments, the calcined catalysts were outgassed under vacuum for 4 h, then for 0.5 h at 80 °C, followed by a period of 3 h at 300 °C. From the isotherms, specific surface areas were calculated using the BET method, micropore volumes and external surface areas were calculated using the *t*-plot method, and mesopore volumes were calculated by difference with the total adsorbed volume.

The density of Brønsted acid sites was determined by *n*-propylamine titration by following the TPD-catalyzed Hoffman elimination in a homemade packed-bed setup connected to a Pfeiffer Omnistar quadrupole mass spectrometer. First, the sample (*ca.* 100 mg) was pretreated at 550 °C for 1 h using a temperature ramp of 10 °C min^–1^ (as-made samples for *in situ* calcination were also tested following the calcination protocol described above). After cooling the samples down to 130 °C, saturated *n*-propylamine in N_2_ (50 cm^3^ min^–1^) was flowed for 1 h through the catalyst to chemisorb the probe molecule. Physisorbed *n*-propylamine was removed by flowing pure N_2_ for 4 h, and then, the temperature was increased up to 650 °C using a temperature ramp of 20 °C min^–1^. Propene and NH_3_ were monitored as the main products from the Hoffman elimination.

Samples for the solid-state nuclear magnetic resonance (NMR) experiments were ground in a mortar and transferred to a 3.2 mm zirconia rotor. ^31^P MAS NMR experiments were performed on a Bruker 400 MHz (9.4 T) wide-bore magnet with an AVANCE-III console equipped with a Bruker 3.2 mm HXY MAS probe. The experiments were performed at room temperature with a MAS frequency of 20 kHz. π/2 pulses were applied with a field strength of 62.5 kHz, a 30 s recycle delay, and an accumulation of 384 scans. ^31^P chemical shifts were referenced externally to ammonium dihydrogen phosphate ((NH_4_)H_2_PO_4_). ^27^Al MAS NMR experiments were performed on a Bruker 900 MHz (21.1 T) wide-bore magnet with an AVANCE-III console equipped with a Bruker 3.2 mm HXY MAS probe. The 1D ^27^Al direct-excitation MAS NMR spectrum was recorded using hard π/6 pulses with a 0.5 s recycle delay, 33 ms acquisition time, and accumulation of 4096 scans at a MAS frequency of 20 kHz. ^27^Al chemical shifts were referenced externally to aluminum chloride hexahydrate (AlCl_3_·6H_2_O).

Water adsorption experiments were carried out at 25 °C in a Vstar of Quantachrome Instruments. The system was designed for subatmospheric pressure measurements by the Advanced Materials Laboratory group at the University of Alicante. Samples were degassed for 4 h at 250 °C prior to each isotherm measurement. He was used to estimate the dead volume, assuming that it is not adsorbed by any of the samples. After water adsorption experiments, the porous texture and crystallinity of the samples were characterized by means of N_2_ adsorption–desorption, CO_2_ adsorption, and XRD. Prior to the adsorption experiments, samples were outgassed at 150 °C for 4 h. The N_2_ adsorption–desorption isotherms were measured at −196 °C and the CO_2_ adsorption at 0 °C in a Quadrawin (Quantachrome) device. P-XRD patterns were collected *in situ* during a calcination in air after water adsorption in Panalytical Empyrean multifunctional equipment. In its basic configuration, the equipment has a goniometer with an X-ray tube with a Cu Kα cathode and a PIXcel 3D detector equipped with a hot chamber (the hot chamber can work up to 900 °C and with a controlled atmosphere). Temperature was raised from room temperature to 500 °C and then cooled down to 25 °C, where a last XRD pattern was recorded.

### Computational Methods

SAPO-18 and MgAPO-18 unit cells (Figure S23) were fully optimized within the density functional theory (DFT) construct as implemented in the Vienna *ab initio* software package^58^ using the generalized gradient approximation functional PBE^59^ and Grimme’s D3 dispersion correction^60^ with the Becke–Johnson damping function.^61^ A projector-augmented-wave plane-wave basis^62^ set was employed with a 500 eV cutoff. Brillouin zone sampling occurred at the γ point. The electronic convergence criterion was set to 10^–6^ eV for electronic energies, and geometries were optimized until forces on the atoms were below 0.01 eV/A.

The DFT-optimized lattice parameters were then adopted for *ab initio* molecular dynamics (AIMD) simulations conducted in the NVT ensemble at the revPBE-D3(BJ)^60,61,63^ level of theory. AIMD simulations were performed with the CP2K^64^ software using the QuickStep^65^ module at a temperature of 400 °C controlled by a Nose–Hoover chain thermostat with five beads and a time constant of 100 fs. The Gaussian and plane-wave (GPW) method^66^ was used with the TZVP basis set for all atoms, and the TZVP-MOLOPT-SR-GTH basis set was used for Al and Mg. Core electrons were represented with GTH pseudopotentials,^67^ and an auxiliary plane-wave cutoff of 400 Ry was used. A 30 ps production run was executed following a 5 ps equilibration run using a time step of 0.5 fs. The dipole moments from the resulting trajectories were calculated every 2 fs using the Berry phase approach.^68^ IR spectra were then calculated as the Fourier transform of the dipole autocorrelation function.^69,70^ A Lorentzian function was then applied to smooth the spectrum with a full width at half-maximum of 6 cm^–1^. Simulating IR frequencies in this manner takes into account anharmonicities and finite temperature effects to provide time-averaged frequencies for the chemical system. However, the output frequencies cannot be separated into their atomistic contributions. Hence, power spectra that isolate the contributions of the molecular guests were additionally generated using TRAVIS^71^ to analyze the same trajectories with a correlation depth of 5 ps.

The simulated IR spectra are dominated by signals originating from the bonds within the zeolitic framework. However, the characteristic frequencies for molecular guests are visible at low absorbances. To clarify the signals originating from confined guest species, molecular power spectra with contributions from the zeolite subtracted are overlaid with the simulated IR spectra. For surface species, including the BAS and its transformations (methoxy and aldehyde species), the presented power spectrum is the global power spectrum, including signals originating from the entire framework. However, the characteristic features are more pronounced because there is less noise in the power spectra. The dipole autocorrelation function used to compute IR spectra is based on the Berry phase approach, which provides the total dipole moment of the simulated system. The complexity of capturing the adiabatic evolution of the entire system can introduce noise into the spectrum. In contrast, the power spectra computed by TRAVIS utilize atomic velocity vectors from AIMD trajectories and zero padding, which provides better resolution.^71^

## Supporting Information Available

Definitions of the reaction indexes, basic characterization of the catalysts (Figure S1 and Tables S1, S3, and S4), additional CO_2_ hydrogenation catalytic tests (Figures S1–S9), temporal analysis of products results (Figure S10), ^13^C NMR experiments (Figure S11), methanol-to-olefins tests (Figures S12–S14), additional XRD analysis and refinements (Figures S15–S16, S18, S22, and Table S2), ^31^P- and ^27^Al-NMR of the catalysts (Figures S17 and S19), reaction–regeneration cycles (Figures S20–S21), and computational details with additional results (Figures S23–S25) (PDF)

## References

[ref1] Zhou W.; Cheng K.; Kang J.; Zhou C.; Subramanian V.; Zhang Q.; Wang Y. New Horizon in C1 Chemistry: Breaking the Selectivity Limitation in Transformation of Syngas and Hydrogenation of CO_2_ into Hydrocarbon Chemicals and Fuels. Chem. Soc. Rev. 2019, 48, 3193–3228. 10.1039/C8CS00502H. 31106785

[ref2] Pan X.; Jiao F.; Miao D.; Bao X. Oxide-Zeolite-Based Composite Catalyst Concept That Enables Syngas Chemistry beyond Fischer–Tropsch Synthesis. Chem. Rev. 2021, 121, 6588–6609. 10.1021/acs.chemrev.0c01012. 34032417

[ref3] Xie J.; Olsbye U. The Oxygenate-Mediated Conversion of COx to Hydrocarbons - On the Role of Zeolites in Tandem Catalysis. Chem. Rev. 2023, 123, 11775–11816. 10.1021/acs.chemrev.3c00058. 37769023 PMC10603784

[ref4] Arora S. S.; Shi Z.; Bhan A. Mechanistic Basis for Effects of High-Pressure H_2_ Cofeeds on Methanol-to-Hydrocarbons Catalysis over Zeolites. ACS Catal. 2019, 9, 6407–6414. 10.1021/acscatal.9b00969.

[ref5] Shi Z.; Bhan A. The Effects of CO Co-Feed on the Catalytic Performance of Methanol-to-Hydrocarbons Conversion on HZSM-5. Chem. Eng. J. 2023, 456, 140867 10.1016/j.cej.2022.140867.

[ref6] Zhao X.; Li J.; Tian P.; Wang L.; Li X.; Lin S.; Guo X.; Liu Z. Achieving a Superlong Lifetime in the Zeolite-Catalyzed MTO Reaction under High Pressure: Synergistic Effect of Hydrogen and Water. ACS Catal. 2019, 9, 3017–3025. 10.1021/acscatal.8b04402.

[ref7] Deluca M.; Kravchenko P.; Hoffman A.; Hibbitts D. Mechanism and Kinetics of Methylating C_6_-C_12_ Methylbenzenes with Methanol and Dimethyl Ether in H-MFI Zeolites. ACS Catal. 2019, 9, 6444–6460. 10.1021/acscatal.9b00650.

[ref8] Cheng K.; Gu B.; Liu X.; Kang J.; Zhang Q.; Wang Y. Direct and Highly Selective Conversion of Synthesis Gas into Lower Olefins: Design of a Bifunctional Catalyst Combining Methanol Synthesis and Carbon-Carbon Coupling. Angew. Chem., Int. Ed. 2016, 55, 4725–4728. 10.1002/anie.201601208. 26961855

[ref9] Jiao F.; Li J.; Pan X.; Xiao J.; Li H.; Ma H.; Wei M.; Pan Y.; Zhou Z.; Li M.; Miao S.; Li J.; Zhu Y.; Xiao D.; He T.; Yang J.; Qi F.; Fu Q.; Bao X. Selective Conversion of Syngas to Light Olefins. Science 2016, 351 (6277), 1065–1068. 10.1126/science.aaf1835. 26941314

[ref10] Ramirez A.; Ticali P.; Salusso D.; Cordero-Lanzac T.; Ould-Chikh S.; Ahoba-Sam C.; Bugaev A. L.; Borfecchia E.; Morandi S.; Signorile M.; Bordiga S.; Gascon J.; Olsbye U. Multifunctional Catalyst Combination for the Direct Conversion of CO_2_ to Propane. JACS Au 2021, 1, 1719–1732. 10.1021/jacsau.1c00302. 34723275 PMC8549042

[ref11] Ticali P.; Salusso D.; Ahmad R.; Ahoba-Sam C.; Ramirez A.; Shterk G.; Lomachenko K. A.; Borfecchia E.; Morandi S.; Cavallo L.; Gascon J.; Bordiga S.; Olsbye U. CO_2_ Hydrogenation to Methanol and Hydrocarbons over Bifunctional Zn-Doped ZrO_2_/Zeolite Catalysts. Catal. Sci. Technol. 2021, 11, 1249–1268. 10.1039/D0CY01550D.

[ref12] Ji Y.; Gao P.; Zhao Z.; Xiao D.; Han Q.; Chen H.; Gong K.; Chen K.; Han X.; Bao X.; Hou G. Oxygenate-Based Routes Regulate Syngas Conversion over Oxide–Zeolite Bifunctional Catalysts. Nat. Catal. 2022, 5, 594–604. 10.1038/s41929-022-00806-2.

[ref13] Su J.; Zhou H.; Liu S.; Wang C.; Jiao W.; Wang Y.; Liu C.; Ye Y.; Zhang L.; Zhao Y.; Liu H.; Wang D.; Yang W.; Xie Z.; He M. Syngas to Light Olefins Conversion with High Olefin/Paraffin Ratio Using ZnCrOx /AlPO-18 Bifunctional Catalysts. Nat. Commun. 2019, 10, 1297 10.1038/s41467-019-09336-1. 30899003 PMC6428864

[ref14] Zhang W.; Wang S.; Guo S.; Qin Z.; Dong M.; Wang J.; Fan W. Effective Conversion of CO_2_ into Light Olefins over a Bifunctional Catalyst Consisting of La-Modified ZnZrOx Oxide and Acidic Zeolite. Catal. Sci. Technol. 2022, 12, 2566–2577. 10.1039/D2CY00210H.

[ref15] Xie J.; Firth D. S.; Cordero-Lanzac T.; Airi A.; Negri C.; Øien-Ødegaard S.; Lillerud K. P.; Bordiga S.; Olsbye U. MAPO-18 Catalysts for the Methanol to Olefins Process: Influence of Catalyst Acidity in a High-Pressure Syngas (CO and H_2_) Environment. ACS Catal. 2022, 12, 1520–1531. 10.1021/acscatal.1c04694. 35096471 PMC8788383

[ref16] Olsbye U.; Svelle S.; Bjorgen M.; Beato P.; Janssens T. V. W.; Joensen F.; Bordiga S.; Lillerud K. P. Conversion of Methanol to Hydrocarbons: How Zeolite Cavity and Pore Size Controls Product Selectivity. Angew. Chem., Int. Ed. 2012, 51, 5810–5831. 10.1002/anie.201103657. 22511469

[ref17] Li G.; Jiao F.; Pan X.; Li N.; Miao D.; Li L.; Bao X. Role of SAPO-18 Acidity in Direct Syngas Conversion to Light Olefins. ACS Catal. 2020, 10, 12370–12375. 10.1021/acscatal.0c03257.

[ref18] Airi A.; Damin A.; Xie J.; Olsbye U.; Bordiga S. Catalyst Sites and Active Species in the Early Stages of MTO Conversion over Cobalt AlPO-18 Followed by IR Spectroscopy. Catal. Sci. Technol. 2022, 12, 2775 10.1039/D2CY00303A.

[ref19] Mortén M.; Mentel Ł.; Lazzarini A.; Pankin I. A.; Lamberti C.; Bordiga S.; Crocellà V.; Svelle S.; Lillerud K. P.; Olsbye U. A Systematic Study of Isomorphically Substituted H-MAlPO-5 Materials for the Methanol-to-Hydrocarbons Reaction. ChemPhysChem 2018, 19, 484–495. 10.1002/cphc.201701024. 29250897 PMC5838544

[ref20] Wang C. M.; Brogaard R. Y.; Weckhuysen B. M.; Nørskov J. K.; Studt F. Reactivity Descriptor in Solid Acid Catalysis: Predicting Turnover Frequencies for Propene Methylation in Zeotypes. J. Phys. Chem. Lett. 2014, 5, 1516–1521. 10.1021/jz500482z. 26270089

[ref21] Brogaard R. Y.; Wang C. M.; Studt F. Methanol-Alkene Reactions in Zeotype Acid Catalysts: Insights from a Descriptor-Based Approach and Microkinetic Modeling. ACS Catal. 2014, 4, 4504–4509. 10.1021/cs5014267.

[ref22] Wang C. M.; Brogaard R. Y.; Xie Z. K.; Studt F. Transition-State Scaling Relations in Zeolite Catalysis: Influence of Framework Topology and Acid-Site Reactivity. Catal. Sci. Technol. 2015, 5, 2814–2820. 10.1039/C4CY01692K.

[ref23] Mortén M.; Cordero-Lanzac T.; Cnudde P.; Redekop E. A.; Svelle S.; Van Speybroeck V.; Olsbye U. Acidity Effect on Benzene Methylation Kinetics over Substituted H-MeAlPO-5 Catalysts. J. Catal. 2021, 404, 594–606. 10.1016/j.jcat.2021.11.002.

[ref24] Yarulina I.; De Wispelaere K.; Bailleul S.; Goetze J.; Radersma M.; Abou-Hamad E.; Vollmer I.; Goesten M.; Mezari B.; Hensen E. J. M.; Martínez-Espín J. S.; Morten M.; Mitchell S.; Perez-Ramirez J.; Olsbye U.; Weckhuysen B. M.; Van Speybroeck V.; Kapteijn F.; Gascon J. Structure–Performance Descriptors and the Role of Lewis Acidity in the Methanol-to-Propylene Process. Nat. Chem. 2018, 10, 804–812. 10.1038/s41557-018-0081-0. 29941905

[ref25] Wang J.; Li G.; Li Z.; Tang C.; Feng Z.; An H.; Liu H.; Liu T.; Li C. A Highly Selective and Stable ZnO-ZrO_2_ Solid Solution Catalyst for CO_2_ Hydrogenation to Methanol. Sci. Adv. 2017, 3, e1701290 10.1126/sciadv.1701290. 28989964 PMC5630239

[ref26] Kattel S.; Ramírez P. J.; Chen J. G.; Rodríguez J. A.; Liu P. Active Sites for CO_2_ Hydrogenation to Methanol on Cu/ZnO Catalysts. Science 2017, 355, 1296–1299. 10.1126/science.aal3573. 28336665

[ref27] De Wispelaere K.; Wondergem C. S.; Ensing B.; Hemelsoet K.; Meijer E. J.; Weckhuysen B. M.; Van Speybroeck V.; Ruiz-Martínez J. Insight into the Effect of Water on the Methanol-to-Olefins Conversion in H-SAPO-34 from Molecular Simulations and in Situ Microspectroscopy. ACS Catal. 2016, 6, 1991–2002. 10.1021/acscatal.5b02139.

[ref28] Bordiga S.; Regli L.; Lamberti C.; Zecchina A.; et al. FTIR Adsorption Studies of H_2_O and CH_3_OH in the Isostructural H-SSZ-13 and H-SAPO-34: Formation of H-Bonded Adducts and Protonated Clusters. J. Phys. Chem. B 2005, 109, 7724–7732. 10.1021/jp044324b. 16851897

[ref29] Grifoni E.; Piccini G. M.; Lercher J. A.; Glezakou V. A.; Rousseau R.; Parrinello M. Confinement Effects and Acid Strength in Zeolites. Nat. Commun. 2021, 12, 2630 10.1038/s41467-021-22936-0. 33976197 PMC8113345

[ref30] Hack J. H.; Dombrowski J. P.; Ma X.; Chen Y.; Lewis N. H. C.; Carpenter W. B.; Li C.; Voth G. A.; Kung H. H.; Tokmakoff A. Structural Characterization of Protonated Water Clusters Confined in HZSM-5 Zeolites. J. Am. Chem. Soc. 2021, 143, 10203–10213. 10.1021/jacs.1c03205. 34210123

[ref31] Valecillos J.; Hita I.; Sastre E.; Aguayo A. T.; Castaño P. Implications of Co-Feeding Water on the Growth Mechanisms of Retained Species on a SAPO-18 Catalyst during the Methanol-to-Olefins Reaction. ChemCatChem 2021, 13, 3140–3154. 10.1002/cctc.202100124.

[ref32] Bollini P.; Chen T. T.; Neurock M.; Bhan A. Mechanistic Role of Water in HSSZ-13 Catalyzed Methanol-to-Olefins Conversion. Catal. Sci. Technol. 2019, 9, 4374–4383. 10.1039/C9CY01015G.

[ref33] Portillo A.; Parra O.; Ereña J.; Aguayo A. T.; Bilbao J.; Ateka A. Effect of Water and Methanol Concentration in the Feed on the Deactivation of In_2_O_3_-ZrO_2_/SAPO-34 Catalyst in the Conversion of CO2/CO to Olefins by Hydrogenation. Fuel 2023, 346, 128298 10.1016/j.fuel.2023.128298.

[ref34] Li B.; Huang P.; Cao P.; Gao W.; Zheng W.; Lian C.; Sun W.; Zhao L. Understanding the Structural Properties of Zeolites for Isobutane Alkylation Based on Adsorption/Diffusion Behaviors. Microporous Mesoporous Mater. 2022, 341, 112040 10.1016/j.micromeso.2022.112040.

[ref35] Omojola T.; Cherkasov N.; McNab A. I.; Lukyanov D. B.; Anderson J. A.; Rebrov E. V.; Van Veen A. C. Mechanistic Insights into the Desorption of Methanol and Dimethyl Ether Over ZSM-5 Catalysts. Catal. Lett. 2018, 148, 474–488. 10.1007/s10562-017-2249-4.

[ref36] Redekop E. A.; Lazzarini A.; Bordiga S.; Olsbye U. A Temporal Analysis of Products (TAP) Study of C_2_-C_4_ Alkene Reactions with a Well-Defined Pool of Methylating Species on ZSM-22 Zeolite. J. Catal. 2020, 385, 300–312. 10.1016/j.jcat.2020.03.020.

[ref37] Chowdhury A. D.; Gascon J. The Curious Case of Ketene in Zeolite Chemistry and Catalysis. Angew. Chem., Int. Ed. 2018, 57, 14982–14985. 10.1002/anie.201808480. 30328242

[ref38] Plessow P. N.; Smith A.; Tischer S.; Studt F. Identification of the Reaction Sequence of the MTO Initiation Mechanism Using Ab Initio-Based Kinetics. J. Am. Chem. Soc. 2019, 141, 5908–5915. 10.1021/jacs.9b00585. 30920821

[ref39] Huber P.; Plessow P. N. The Role of Decarboxylation Reactions during the Initiation of the Methanol-to-Olefins Process. J. Catal. 2023, 428, 115134 10.1016/j.jcat.2023.115134.

[ref40] Chen W.; Li G.; Yi X.; Day S. J.; Tarach K. A.; Liu Z.; Liu S. B.; Edman Tsang S. C.; Góra-Marek K.; Zheng A. Molecular Understanding of the Catalytic Consequence of Ketene Intermediates under Confinement. J. Am. Chem. Soc. 2021, 143, 15440–15452. 10.1021/jacs.1c08036. 34478267 PMC8461653

[ref41] Liu Y.; Kirchberger F. M.; Müller S.; Eder M.; Tonigold M.; Sanchez-Sanchez M.; Lercher J. A. Critical Role of Formaldehyde during Methanol Conversion to Hydrocarbons. Nat. Commun. 2019, 10, 1462 10.1038/s41467-019-09449-7. 30931945 PMC6443648

[ref42] Hwang A.; Bhan A. Deactivation of Zeolites and Zeotypes in Methanol-to-Hydrocarbons Catalysis: Mechanisms and Circumvention. Acc. Chem. Res. 2019, 52, 2647–2656. 10.1021/acs.accounts.9b00204. 31403774

[ref43] Colthup N. B.; Daly L. H.; Wiberley S. E. Introduction to Infrared and Raman Spectroscopy; Academic Press, 1975.

[ref44] Ramirez A.; Gong X.; Caglayan M.; Nastase S. A. F.; Abou-Hamad E.; Gevers L.; Cavallo L.; Dutta Chowdhury A.; Gascon J. Selectivity Descriptors for the Direct Hydrogenation of CO_2_ to Hydrocarbons during Zeolite-Mediated Bifunctional Catalysis. Nat. Commun. 2021, 12, 5974 10.1038/s41467-021-26090-5. 34625554 PMC8501036

[ref45] Çağlayan M.; Lucini Paioni A.; Abou-Hamad E.; Shterk G.; Pustovarenko A.; Baldus M.; Chowdhury A. D.; Gascon J. Initial Carbon–Carbon Bond Formation during the Early Stages of Methane Dehydroaromatization. Angew. Chem., Int. Ed. 2020, 59, 16741–16746. 10.1002/anie.202007283. 32521078

[ref46] Fu D.; Lucini Paioni A.; Lian C.; van der Heijden O.; Baldus M.; Weckhuysen B. M. Elucidating Zeolite Channel Geometry–Reaction Intermediate Relationships for the Methanol-to-Hydrocarbon Process. Angew. Chem., Int. Ed. 2020, 59, 20024–20030. 10.1002/anie.202009139. PMC7692936 32761941

[ref47] Ticali P.; Morandi S.; Shterk G.; Ould-Chikh S.; Ramirez A.; Gascon J.; Chung S. H.; Ruiz-Martinez J.; Bordiga S. PdZn/ZrO_2_+SAPO-34 Bifunctional Catalyst for CO_2_ Conversion: Further Insights by Spectroscopic Characterization. Appl. Catal., A 2023, 655, 119100 10.1016/j.apcata.2023.119100.

[ref48] Chen Z.; Ni Y.; Zhi Y.; Wen F.; Zhou Z.; Wei Y.; Zhu W.; Liu Z. Coupling of Methanol and Carbon Monoxide over H-ZSM-5 to Form Aromatics. Angew. Chem., Int. Ed. 2018, 57, 12549–12553. 10.1002/anie.201807814. 30062835

[ref49] Olsbye U.; Svelle S.; Lillerud K. P.; Wei Z. H.; Chen Y. Y.; Li J. F.; Wang J. G.; Fan W. B. The Formation and Degradation of Active Species during Methanol Conversion over Protonated Zeotype Catalysts. Chem. Soc. Rev. 2015, 44, 7155–7176. 10.1039/C5CS00304K. 26185806

[ref50] Simmen A.; McCusker L. B.; Baerlocher C.; Meier W. M. The Structure Determination and Rietveld Refinement of the Aluminophosphate AIPO4–18. Zeolites 1991, 11, 654–661. 10.1016/S0144-2449(05)80167-8.

[ref51] Smith R. L.; Sawiński W. A.; Lind A.; Wragg D. S.; Cavka J. H.; Arstad B.; Fjellvag H.; Attfield M. P.; Akporiaye D.; Anderson M. W. Nanoporous Intergrowths: How Crystal Growth Dictates Phase Composition and Hierarchical Structure in the CHA/AEI System. Chem. Mater. 2015, 27, 4205–4215. 10.1021/cm504284x.

[ref52] Smith R. L.; Svelle S.; Del Campo P.; Fuglerud T.; Arstad B.; Lind A.; Chavan S.; Attfield M. P.; Akporiaye D.; Anderson M. W. CHA/AEI Intergrowth Materials as Catalysts for the Methanol-to-Olefins Process. Appl. Catal., A 2015, 505, 1–7. 10.1016/j.apcata.2015.06.027.

[ref53] Shen B.; Chen X.; Fan X.; Xiong H.; Wang H.; Qian W.; Wang Y.; Wei F. Resolving Atomic SAPO-34/18 Intergrowth Architectures for Methanol Conversion by Identifying Light Atoms and Bonds. Nat. Commun. 2021, 12, 2212 10.1038/s41467-021-22438-z. 33850118 PMC8044160

[ref54] He H.; Klinowski J. Solid State NMR Studies of the Aluminophosphate Molecular Sieve AlPO4–18. J. Phys. Chem. A 1993, 97, 10385–10388. 10.1021/j100142a020.

[ref55] Sławiński W. A.; Wragg D. S.; Akporiaye D.; Fjellvåg H. Intergrowth Structure Modelling in Silicoaluminophosphate SAPO-18/34 Family. Microporous Mesoporous Mater. 2014, 195, 311–318. 10.1016/j.micromeso.2014.04.024.

[ref56] Poulet G.; Tuel A.; Sautet P. A Combined Experimental and Theoretical Evaluation of the Structure of Hydrated Microporous Aluminophosphate AlPO_4_–18. J. Phys. Chem. B 2005, 109, 22939–22946. 10.1021/jp050670x. 16853989

[ref57] Bruce Railsback L. An Earth Scientist’s Periodic Table of the Elements and Their Ions. Geology 2003, 31, 737–740. 10.1130/0016-7606(2005)117<746:AESPTO>2.0.CO;2.

[ref58] Hafner J. Ab-Initio Simulations of Materials Using VASP: Density-Functional Theory and Beyond. J. Comput. Chem. 2008, 29, 2044–2078. 10.1002/jcc.21057. 18623101

[ref59] Perdew J. P.; Burke K.; Ernzerhof M. Generalized Gradient Approximation Made Simple. Phys. Rev. Lett. 1996, 77, 3865–3868. 10.1103/PhysRevLett.77.3865. 10062328

[ref60] Grimme S.; Antony J.; Ehrlich S.; Krieg H. A Consistent and Accurate Ab Initio Parametrization of Density Functional Dispersion Correction (DFT-D) for the 94 Elements H-Pu. J. Chem. Phys. 2010, 132, 154104 10.1063/1.3382344. 20423165

[ref61] Grimme S.; Ehrlich S.; Goerigk L. Effect of the Damping Function in Dispersion Corrected Density Functional Theory. J. Comput. Chem. 2011, 32, 1456–1465. 10.1002/jcc.21759. 21370243

[ref62] Blöchl P. E. Projector Augmented-Wave Method. Phys. Rev. B 1994, 50, 17953–17979. 10.1103/PhysRevB.50.17953. 9976227

[ref63] Hammer B.; Hansen L. B.; Nørskov J. K. Improved Adsorption Energetics within Density-Functional Theory Using Revised Perdew-Burke-Ernzerhof Functionals. Phys. Rev. B 1999, 59, 7413–7421. 10.1103/PhysRevB.59.7413.

[ref64] Kühne T. D.; Iannuzzi M.; Del Ben M.; Rybkin V. V.; Seewald P.; Stein F.; Laino T.; Khaliullin R. Z.; Schütt O.; Schiffmann F.; Golze D.; Wilhelm J.; Chulkov S.; Bani-Hashemian M. H.; Weber V.; Borštnik U.; Taillefumier M.; Jakobovits A. S.; Lazzaro A.; Pabst H.; Müller T.; Schade R.; Guidon M.; Andermatt S.; Holmberg N.; Schenter G. K.; Hehn A.; Bussy A.; Belleflamme F.; Tabacchi G.; Glöß A.; Lass M.; Bethune I.; Mundy C. J.; Plessl C.; Watkins M.; VandeVondele J.; Krack M.; Hutter J. CP2K: An Electronic Structure and Molecular Dynamics Software Package -Quickstep: Efficient and Accurate Electronic Structure Calculations. J. Chem. Phys. 2020, 152, 194103 10.1063/5.0007045. 33687235

[ref65] Vandevondele J.; Krack M.; Mohamed F.; Parrinello M.; Chassaing T.; Hutter J. Quickstep: Fast and Accurate Density Functional Calculations Using a Mixed Gaussian and Plane Waves Approach. Comput. Phys. Commun. 2005, 167, 103–128. 10.1016/j.cpc.2004.12.014.

[ref66] Lippert G.; Hutter J.; Parrinello M. The Gaussian and Augmented-Plane-Wave Density Functional Method for Ab Initio Molecular Dynamics Simulations. Theor. Chem. Acc. 1999, 103, 124–140. 10.1007/s002140050523.

[ref67] Goedecker S.; Teter M.; Hutter J. Separable Dual-Space Gaussian Pseudopotentials. Phys. Rev. B 1996, 54, 1703–1710. 10.1103/PhysRevB.54.1703. 9986014

[ref68] Thomas M.; Brehm M.; Fligg R.; Vöhringer P.; Kirchner B. Computing Vibrational Spectra from Ab Initio Molecular Dynamics. Phys. Chem. Chem. Phys. 2013, 15, 6608–6622. 10.1039/c3cp44302g. 23416970

[ref69] Hoffman A. E. J.; Vanduyfhuys L.; Nevjestić I.; Wieme J.; Rogge S. M. J.; Depauw H.; Van Der Voort P.; Vrielinck H.; Van Speybroeck V. Elucidating the Vibrational Fingerprint of the Flexible Metal-Organic Framework MIL-53(Al) Using a Combined Experimental/Computational Approach. J. Phys. Chem. C 2018, 122, 2734–2746. 10.1021/acs.jpcc.7b11031. PMC5808359 29449906

[ref70] Millan R.; Cnudde P.; Hoffman A. E. J.; Lopes C. W.; Concepción P.; Van Speybroeck V.; Boronat M. Theoretical and Spectroscopic Evidence of the Dynamic Nature of Copper Active Sites in Cu-CHA Catalysts under Selective Catalytic Reduction (NH_3_-SCR-NOx) Conditions. J. Phys. Chem. Lett. 2020, 11, 10060–10066. 10.1021/acs.jpclett.0c03020. 33179925 PMC7720274

[ref71] Brehm M.; Thomas M.; Gehrke S.; Kirchner B. TRAVIS—A Free Analyzer for Trajectories from Molecular Simulation. J. Chem. Phys. 2020, 152, 164105 10.1063/5.0005078. 32357781

