# Supplementary material for: Transitioning from Methanol to Olefins (MTO) toward a Tandem CO2 Hydrogenation Process: On the Role and Fate of Heteroatoms (Mg, Si) in MAPO-18 Zeotypes
Source: JACS Au. 2024 Feb 13;4(2):744–59. doi: 10.1021/jacsau.3c00768 (PMC10900493; doi:10.1021/jacsau.3c00768)
Supplement: Supplementary file 1 — au3c00768_si_001.pdf [file au3c00768_si_001.pdf]

Supporting Information

Transitioning from Methanol-to-Olefins (MTO) towards Tandem CO_2 hydrogenation process: On the role and fate of heteroatoms (Mg, Si) in MAPO-18 zeotypes

Tomas Cordero-Lanzac[1]*, Izar Capel Berdiell[1], Alessia Airi[2], Sang-Ho Chung[3], Jenna L. Mancuso[4], Evgeniy A. Redekop[1], Claudia Fabris[1], Leidy Figueroa-Quintero[5], Juan C. Navarro[3], Javier Narciso[5], Enrique V. Ramos-Fernandez[5], Stian Svelle[1], Veronique van Speybroeck[4], Javier Ruiz-Martinez[3], Silvia Bordiga[2]*, Unni Olsbye[1]*

[1] *SMN Centre for Materials Science and Nanotechnology, Department of Chemistry, University of Oslo, 0371 Oslo, Norway*

[2] *Department of Chemistry, NIS Center and INSTM Reference Center, University of Turin, Turin 10125, Italy*

[3] *KAUST Catalysis Center (KCC), King Abdullah University of Science and Technology, Thuwal, Saudi Arabia*

[4] *Center for Molecular Modeling, Ghent University, Technologiepark 46, B-9052 Zwijnaarde, Belgium*

[5] *Laboratory of Advanced Materials, Inorganic Chemistry Department, University Materials Institute of Alicante, University of Alicante, Apartado 99, Alicante, 03080, Spain*

*corresponding authors: t.c.lanzac@smn.uio.no, unni.olsbye@kjemi.uio.no, and silvia.bordiga@unito.it

Contents

1. Definition of reaction indexes 3

2. Role of Mg and Si heteroatoms in the reactivity 4

3. Stability and fate of heteroatoms 13

4. Computational details 24

References 31

S1. Definition of reaction indexes

The following reaction indexes were defined to monitor the evolution of reactions with time. For the tandem CO_2 hydrogenation to hydrocarbons, CO_2 conversion is defined as:

$$X_{\mathrm{CO_2}} = \frac{F_{prod}}{F_{\mathrm{CO_2},in}} \tag{S1}$$

where F_{prod}, $F_{\mathrm{CO2},in}$ are the molar flow rate in C_1 basis of products at the outlet of the reactor and of CO_2 at the inlet of the reactor, respectively. Oxygenate conversion is defined assuming that all hydrocarbon products were formed from either methanol or dimethyl ether

$$X_{\mathrm{OX}} = \frac{F_{\mathrm{HC}}}{F_{\mathrm{OX}} + F_{\mathrm{HC}}} \tag{S2}$$

where F_{OX} and F_{HC} are the molar flow rate in C_1 basis of hydrocarbons and oxygenates at the outlet of the reactor, respectively. In the reactions with methanol in the feed, the same definition of oxygenate conversion was used. The selectivity index for the tandem CO_2 hydrogenation is defined based on the main reaction products and lumping all hydrocarbons

$$S_i = \frac{F_i}{\sum_i F_i}, \quad i = \mathrm{CO}, \mathrm{OX}, \mathrm{HC} \tag{S3}$$

where F_i is the molar flow rate of CO, oxygenates or hydrocarbons in C_1 basis at the outlet of the reactor. Hydrocarbons distribution in the tandem CO_2 hydrogenation or selectivity in the MTH reaction is defined by considering methanol and DME as reactants

$$S_j = \frac{F_j}{\sum_j F_j}, \quad j = \mathrm{C_1}\text{-}\mathrm{C_{5+}} \text{ hydrocarbons} \tag{S4}$$

where F_j is the molar flow rate of methane, ethane/ethene, propane/propene, butanes/butenes and C_{5+} hydrocarbons in C_1 basis at the outlet of the reactor.

S2. Role of Mg and Si heteroatoms in the reactivity

Catalyst characterization

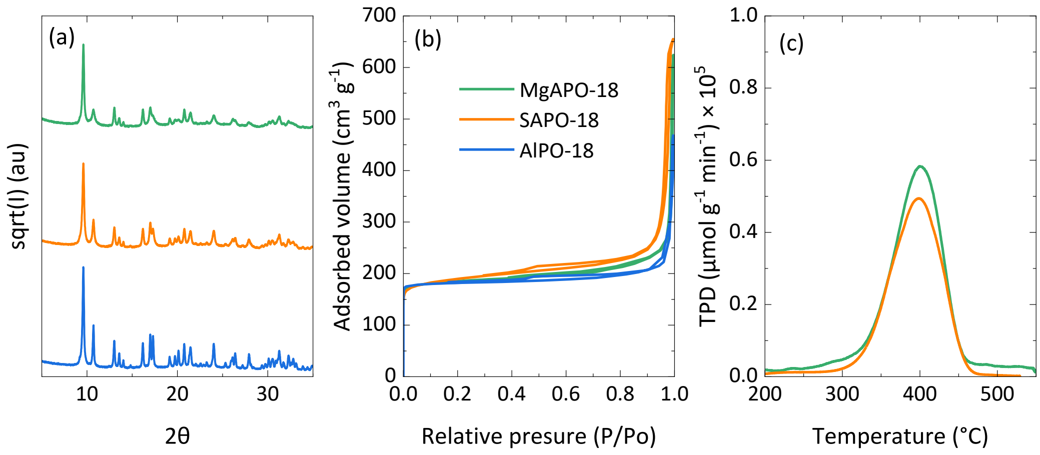

Figure S1. (a) XRD patterns, (b) N_2 adsorption-desorption isotherms and (c) propylamine TPD profile of MgAPO-18, SAPO-18 and AlPO-18 zeotypes. Propene signal was negligible for the AlPO-18 during propylamine TPD.

Table S1. Textural and acid properties of the freshly calcined MgAPO-18, SAPO-18 and AlPO-18 zeotypes.

	S_{BET} (m^2 g^{-1})	S_{ext} (m^2 g^{-1})	V_{mic} (cm^3 g^{-1})	V_{mes} (cm^3 g^{-1})	Density of acid site (mmol g^{-1})
MgAPO-18	742	61	0.26	0.70	0.46
SAPO-18	770	62	0.27	0.61	0.41
AlPO-18	757	36	0.27	0.46	-

Supporting catalytic results

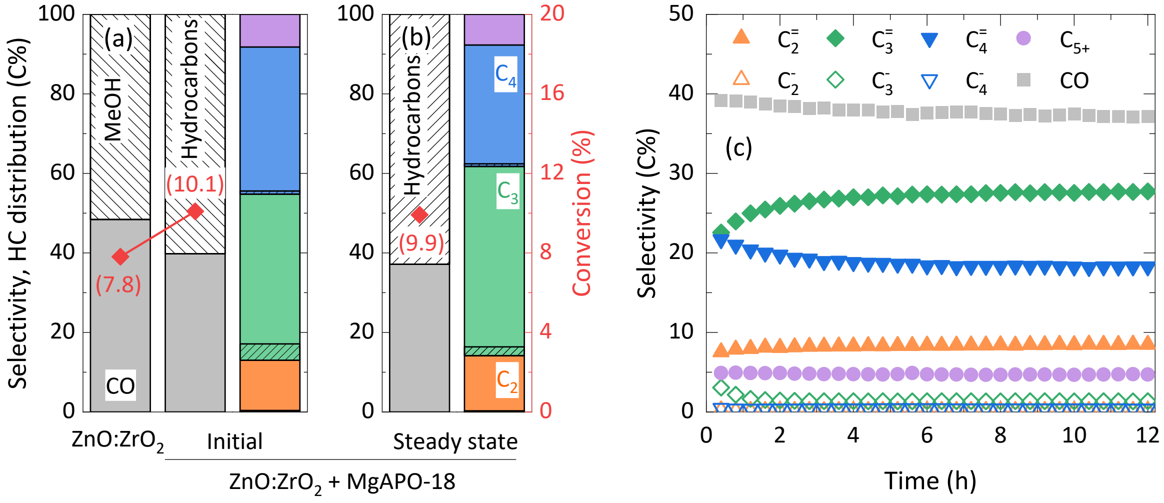

Figure S2. Comparison of (a) the initial and (b) steady-state CO_2 conversion (red symbols), CO selectivity (grey), MeOH or hydrocarbon selectivity (stripped white) and hydrocarbon distribution (colored bars, with striped patterns referring to the amount of paraffins in each hydrocarbon fraction) for the CO_2 hydrogenation to methanol and the tandem CO_2 hydrogenation to hydrocarbons. (b) Evolution with time of product distribution. (ZnO:ZrO_2 + MgAPO-18 catalysts with 1/1 ratio. 375 ºC, 30 bar, 12000 cm^3 h^{-1} g^{-1}, H_2/CO_2 ratio of 3. Samples were calcined and stored before testing.

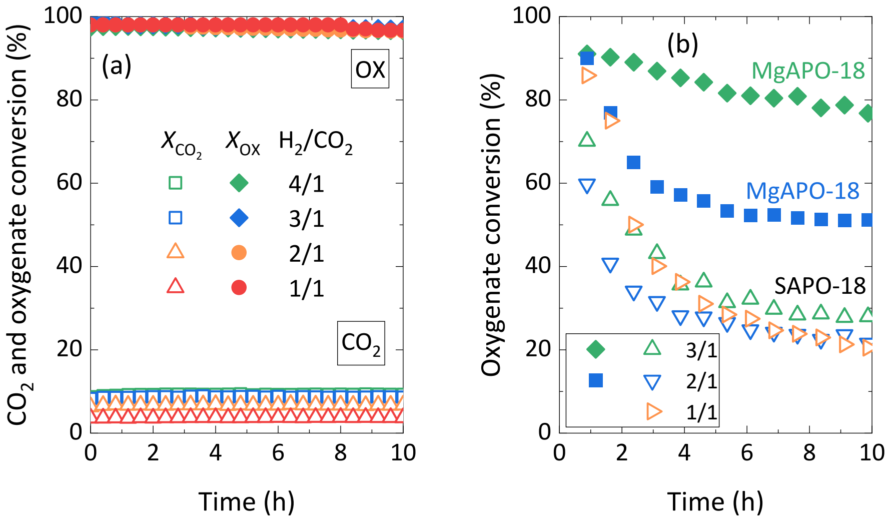

Figure S3. Effect of the H_2/CO_x feed ratio (a) on the CO_2 and oxygenates conversion in the CO_2 tandem hydrogenation to hydrocarbons (350 ºC, 30 bar, 12000 $cm^3\ h^{-1}\ g^{-1}$) using ZnO:ZrO_2 + MgAPO-18 catalysts in a 1/1 ratio and (b) on the oxygenates conversion in the methanol-to-hydrocarbons reaction at CO_2 hydrogenation conditions (350 ºC, 20 bar, 0.5 bar methanol in, 2.5 $g_{MeOH}\ h^{-1}\ g^{-1}$). Samples were calcined and stored before testing.

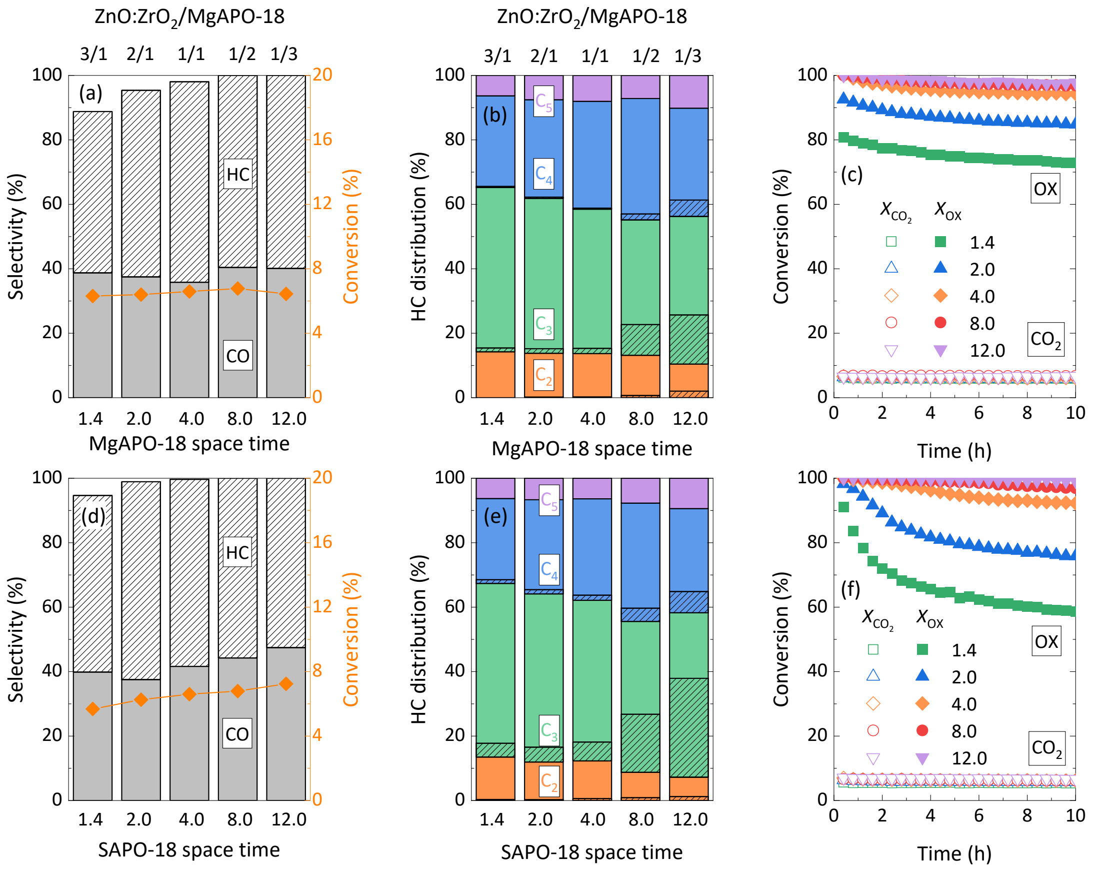

Figure S4. Effect of MAPO-18 amount in the catalytic bed on the initial (a, d) CO_2 conversion, CO and hydrocarbons selectivity and (b, e) hydrocarbon distribution (striped patterns referring to the amount of paraffins in each hydrocarbon fraction) in the CO_2 tandem hydrogenation to hydrocarbons using ZnO:ZrO_2 + (a, b) MgAPO-18 and (d, e) SAPO-18 catalysts. Evolution with time of CO_2 and oxygenates conversion during the first 10 h on stream for the (c) MgAPO-18 and (f) SAPO-18 catalysts. 350 ºC, 30 bar, H_2/CO_2 ratio of 3, fixed CO_2/ZnO:ZrO_2 ratio. ZnO:ZrO_2/MAPO-18 ratios correspond to the studied MAPO-18 space time range in Figure 2. Samples were calcined and stored before testing.

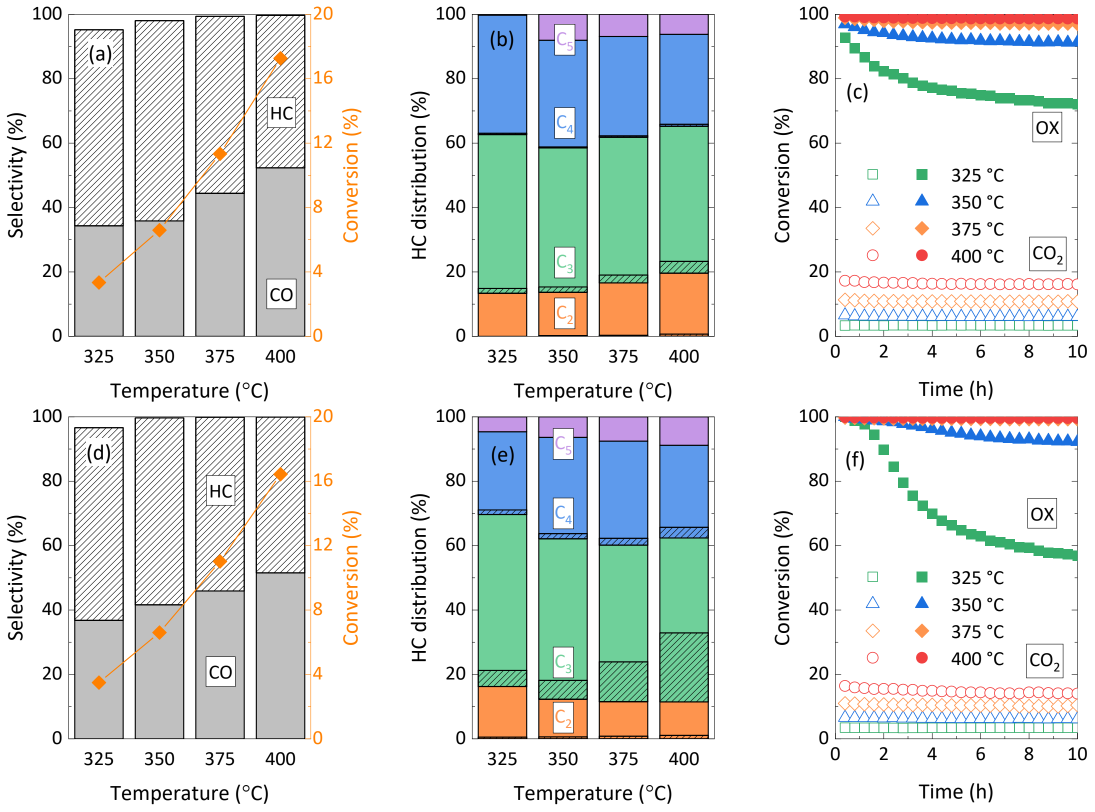

Figure S5. Effect of temperature on the initial (a, d) CO_2 conversion, CO and hydrocarbons selectivity and (b, e) hydrocarbon distribution (striped patterns referring to the amount of paraffins in each hydrocarbon fraction) in the CO_2 tandem hydrogenation to hydrocarbons using $ZnO:ZrO_2$ + (a, b) MgAPO-18 and (d, e) SAPO-18 catalysts in a 1/1 ratio. Evolution with time of CO_2 and oxygenates conversion during the first 10 h on stream for the (c) MgAPO-18 and (f) SAPO-18 catalysts. 30 bar, 12000 cm^3 h^{-1} g^{-1}, H_2/CO_2 ratio of 3. Samples were calcined and stored before testing.

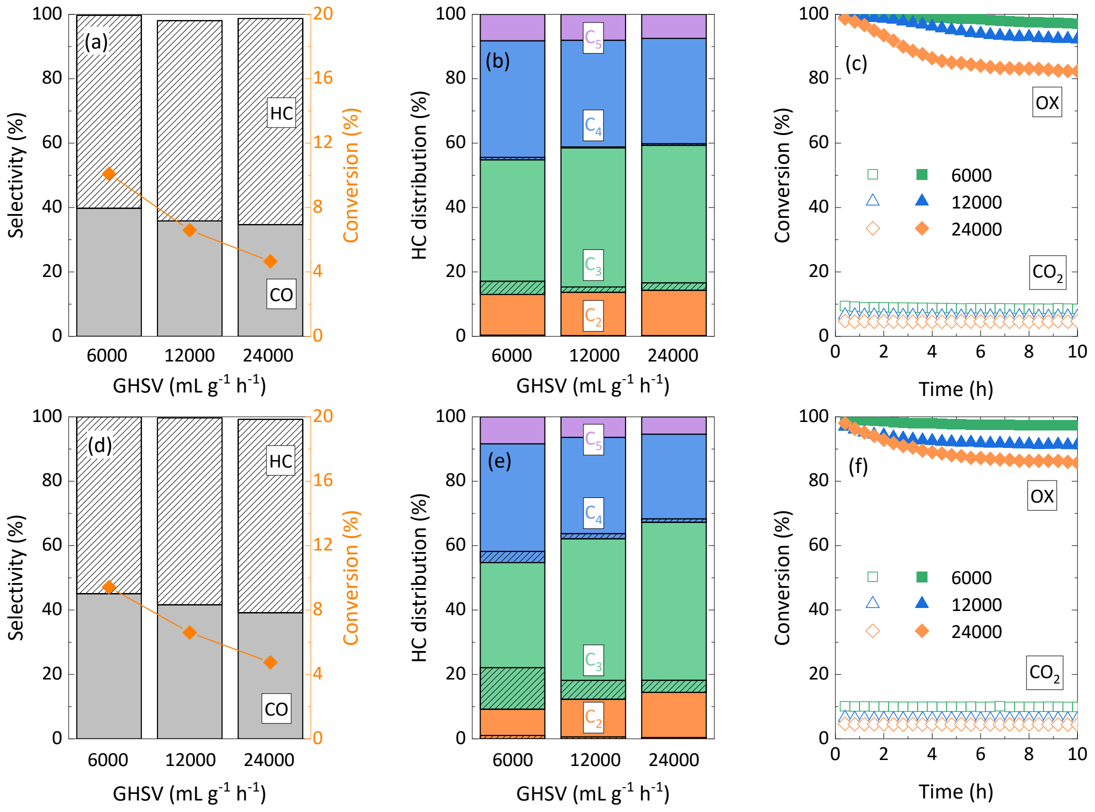

Figure S6. Effect of Gas Hourly Space Velocity (GHSV) on the initial (a, d) CO_2 conversion, CO and hydrocarbons selectivity and (b, e) hydrocarbon distribution (striped patterns referring to the amount of paraffins in each hydrocarbon fraction) in the CO_2 tandem hydrogenation to hydrocarbons using ZnO:ZrO_2 + (a, b) MgAPO-18 and (d, e) SAPO-18 catalysts in a 1/1 ratio. Evolution with time of CO_2 and oxygenates conversion during the first 10 on stream for the (c) MgAPO-18 and (f) SAPO-18 catalysts. 350 ºC, 30 bar, H_2/CO_2 ratio of 3. Samples were calcined and stored before testing.

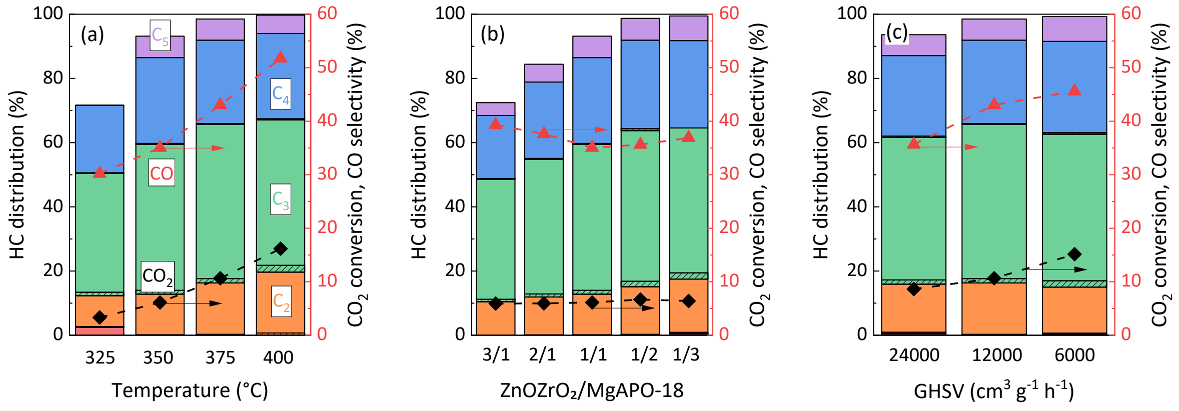

Figure S7. Effect of (a) temperature, (b) $ZnO:ZrO_2$/MgAPO-18 ratio and (c) GHSV on the steady-state CO_2 conversion (black), CO selectivity (red) and hydrocarbon distribution in the CO_2 tandem hydrogenation to hydrocarbons using $ZnO:ZrO_2$ + MgAPO-18. 350 ºC, 30 bar, H_2/CO_2 ratio of 3, Fixed $CO_2/ZnO:ZrO_2$ ratio. Bar values below 100% mean that unconverted methanol has been detected in the effluent. Striped patterns refers to the amount of paraffins in each hydrocarbon fraction. Samples were calcined and stored before testing.

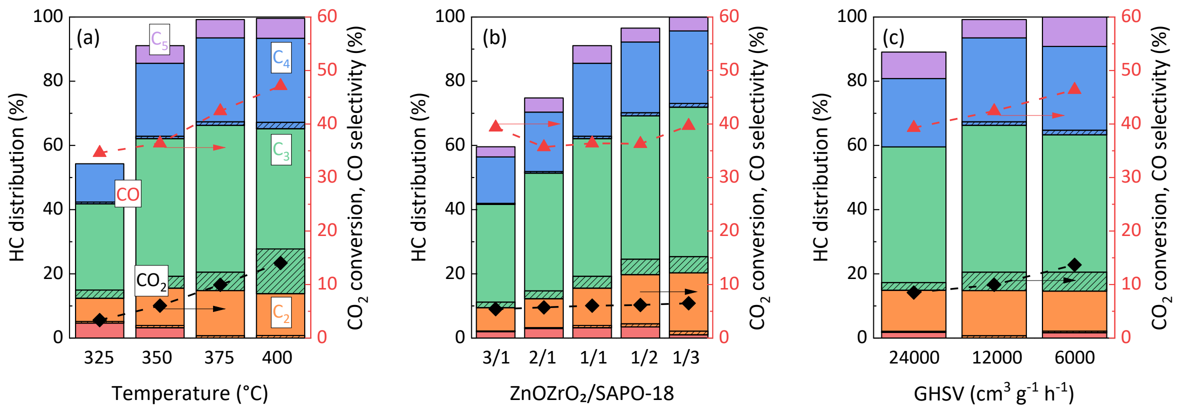

Figure S8. Effect of (a) temperature, (b) $ZnO:ZrO_2$/SAPO-18 ratio and (c) GHSV on the steady-state CO_2 conversion (black), CO selectivity (red) and hydrocarbon distribution in the CO_2 tandem hydrogenation to hydrocarbons using $ZnO:ZrO_2$ + SAPO-18. 350 ºC, 30 bar, H_2/CO_2 ratio of 3, Fixed $CO_2/ZnO:ZrO_2$ ratio. Bar values below 100% mean that unconverted methanol has been detected in the effluent. Striped patterns refers to the amount of paraffins in each hydrocarbon fraction. Samples were calcined and stored before testing.

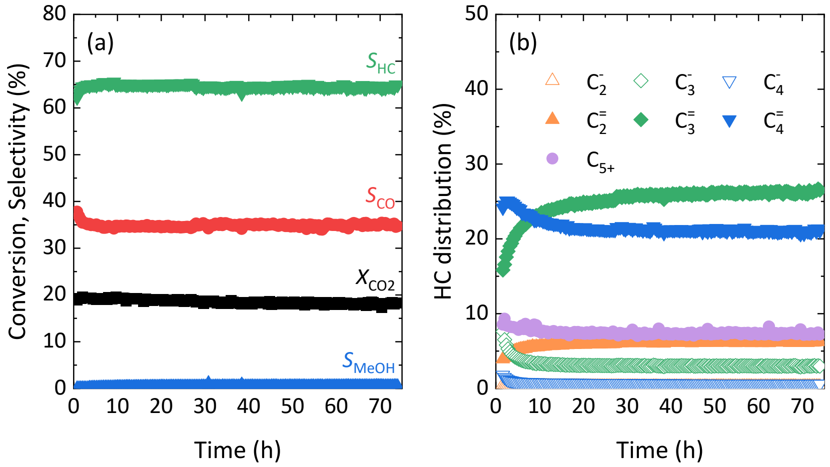

Figure S9. Evolution with time on stream of (a) CO_2 conversion and selectivities to CO methanol and hydrocarbon and (c) hydrocarbon distribution. ZnO:ZrO_2 + MgAPO-18 catalysts with 1/1 ratio. 350 ºC, 30 bar, 2000 cm^3 h^{-1} g^{-1}, H_2/CO_2 ratio of 3.

Temporal Analysis of Products (TAP) experiments

Temporal Analysis of Products (TAP) pulse-response experiments were conducted using a TAP-3E Reactor System from Mithra Tech., following the protocol described in details elsewhere.[1] In brief, 1 nmol pulses of 25% mixture of each hydrocarbon (C_3H_6 3.5 Praxair, i-C_4H_8 2.0 Praxair) with Ne (4.5 Linde) were injected at different temperatures into an evacuated (10^{-8} mbar background pressure) tubular packed-bed reactor (50 mm total length, 4 mm inner diameter) containing a thin (ca. 2 mm) layer of pelletized (200 < dp < 400 µm) MAPO-18 sample (the rest of the reactor packed with quartz (Merck) particles of the same sieve fraction). The reactor effluent was monitored with a calibrated Extrel Quadrupole Mass-Spectrometer at 1 ms time resolution. For the final analysis, signals from consecutive pulses were averaged (10 pulses per amu) to reduce signal to noise ratio.

The conditions of high vacuum of TAP (10^{-8}-10^{-6} mbar) allows decoupling diffusion and adsorption events from reaction and provides valuable information on the pure interaction between reactant molecules and active sites. Propylene and isobutene were used as probe molecules, and a neutral AlPO-18 was used for blank tests. This material was synthesized following a similar protocol and characterization results can be found in **Figure S3** in **Table S1**. The fast responses of propene and isobutene (blue lines in **Figure 3a** and **3b**, respectively) observed with the neutral AlPO-18 confirm the negligible interaction of reactants with this material due to the absence of acid sites inside the channels or on the external surface. Therefore, this response shape can be used as a transport standard all studied materials. The pulse response for propene over the SAPO-18 shows only a slightly longer tale in the 1 s range, whereas a significant delay in the propene response is observed for the MgAPO-18 (**Figure S10a**). Intrinsically, isobutene interacts stronger than propene with the acid sites due to higher stability of the tertiary protonated carbocation. However, a sharper response is observed for isobutene over MgAPO-18 than propene over the same material and isobutene over SAPO-18, suggesting that the number of external acid sites is significantly higher on SAPO-18.

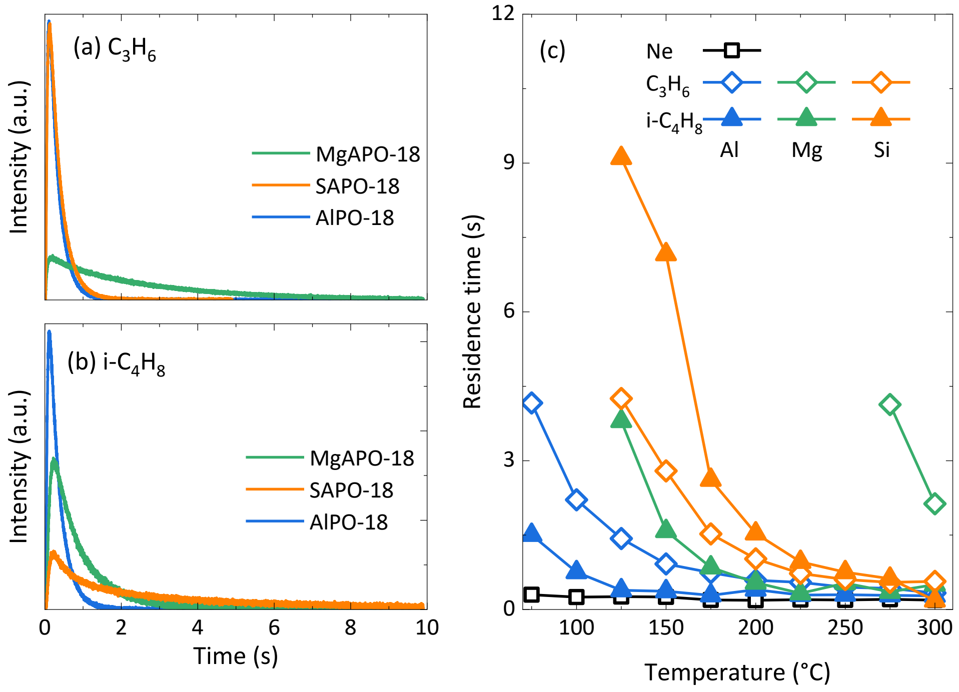

Figure S10. Temporal analysis response of (a) propene at 275 ºC and (b) isobutene at 150 ºC, and (c) evolution with temperature of mass-corrected mean residence times of propene and isobutene over MAPO-18 (M = Mg, Si) zeotypes. Results with non-acid AlPO-18 included for comparison. Samples were calcined and stored before testing.

^{1}H and ^{13}C solid state NMR

All ^{1}H and ^{13}C related (both 1D and 2D) MAS ssNMR spectroscopic experiments were performed on Bruker AVANCE III spectrometers operating at 600 MHz (14.1 T) using a 3.2 mm HXY MAS probe. Herein, samples were pretreated at 550 °C in synthetic air condition (30% oxygen and 70% nitrogen, 30 mL/min of total flow) with the heating ramp of 5 °C for 1 h and were cooled down to 350 °C under nitrogen. Then, to increase the NMR sensitivity, the samples were prepared using enriched ^{13}C methanol ($^{13}CH_3OH$, Cortecnet) for 30 min at experimental conditions (1 bar, 350 °C, WHSV 5.5 h^{-1}). All NMR measurements were performed at room temperature and MAS frequency of 20 kHz. The 1D direct excitation (DE) spectra were recorded using $\pi/2$ pulses of a field strength of 50 kHz and the accumulation of 2048 scans with a 4 s recycle delay. 2D ^{13}C-^{13}C spectra were recorded using a 2 s recycle delay, 10 ms (F2) and 1.3 ms (F1) acquisition time and an accumulation of 256 scans. ^{13}C-^{13}C mixing was achieved through proton driven spin-diffusion (PDSD) using phase-alternated-recoupling-irradiation-schemes (PARIS) for 120 ms (CP). 70 kHz SPINAL64 1H decoupling was applied during both direct and indirect dimensions. 2D ^{1}H-^{13}C INEPT-based heteronuclear correlation (HETCOR) was applied with 1.5 s recycle delay, 17 ms (^{13}C, F2) and 4 ms (^{1}H, F1) acquisition time and an accumulation of 512 scans. ^{1}H and ^{13}C NMR chemical shifts are reported with respect to the external reference adamantane. All NMR spectra were processed and analyzed using Bruker TopSpin version 4.1.4.

One-dimensional (1D) ^{13}C direct excitation (DE) pulse sequence was applied to detect all chemical species in quantitative analysis (**Figure S11a**). Generally, ^{13}C NMR spectra of the post-reacted zeotypes show the following three features: (i) 5-50 ppm (aliphatics), (ii) 110-150 ppm (aromatics), and (iii) 140-300 ppm (carbocations and carbonyls).[2–5]

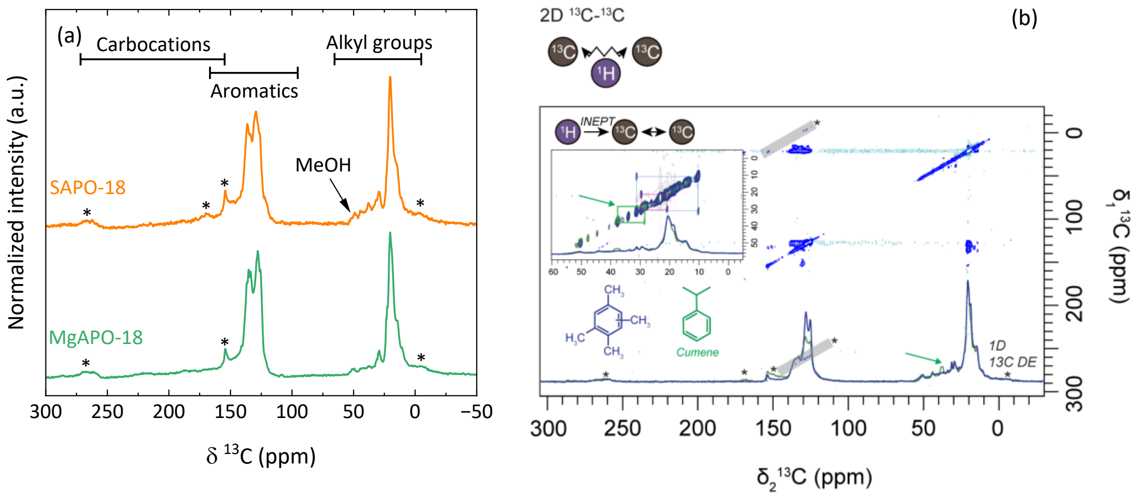

Figure S11. (a) 1D ^{13}C NMR spectra and (b) 2D ^{13}C-^{13}C for in-situ NMR after 30 min on stream of methanol-to-hydrocarbons reaction using ^{13}C-labeled methanol and MAPO-18 (M = Mg, Si) zeotypes. The ratio between aromatic and alkyl groups is estimated 1.24 and 1.22 for SAPO-18 and MgAPO-18 zeotypes, respectively. . The 2D ^{13}C-^{13}C correlation experiments were achieved through proton-driven spin diffusion using phase alternated recoupling irradiations schemes (PDSD PARIS). MAS spinning rate at 20 kHz and the spinning side bands are noted with asterisk.

S3. Stability and fate of heteroatoms

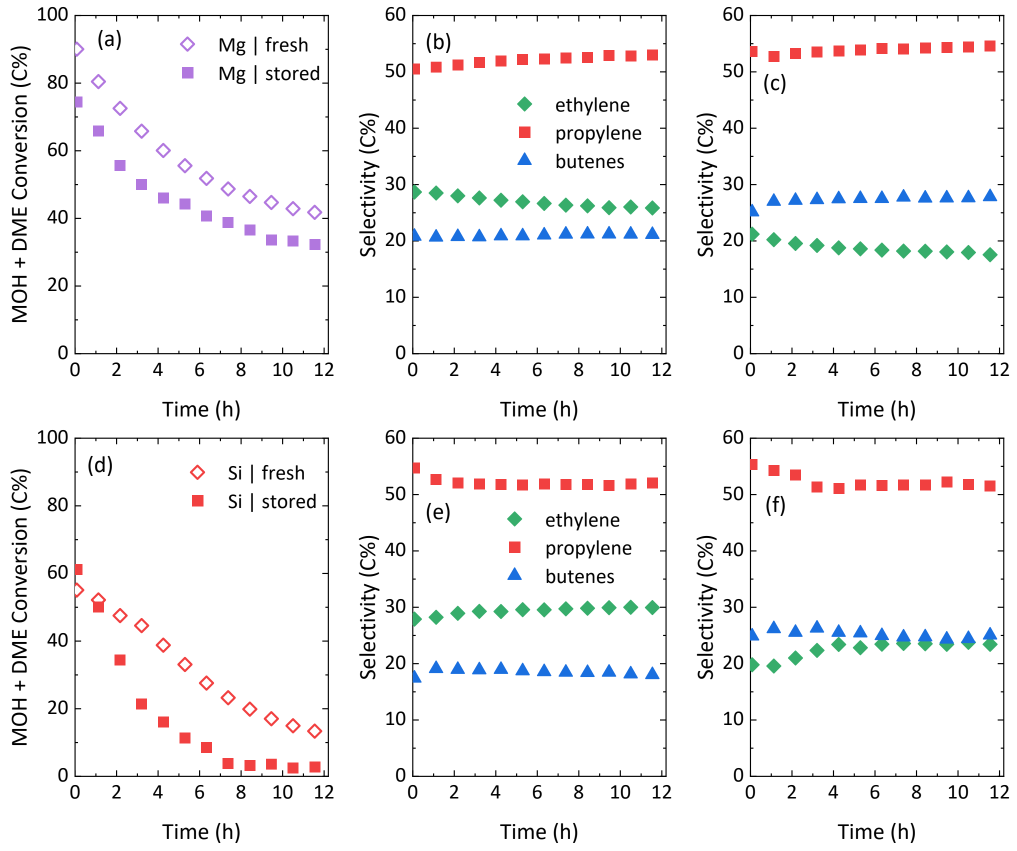

Figure S12. Evolution with time of (a, d) methanol conversion and (b, c, e, f) product distribution in the methanol to hydrocarbons using (b) freshly calcined MgAPO-18, (c) calcined and stored MgAPO-18, (e) freshly calcined SAPO-18 and (f) calcined and stored SAPO-18. 350 ºC, 1 bar, 40 mbar MeOH in He, 6.5 g_{MeOH} h^{-1} g^{-1}.

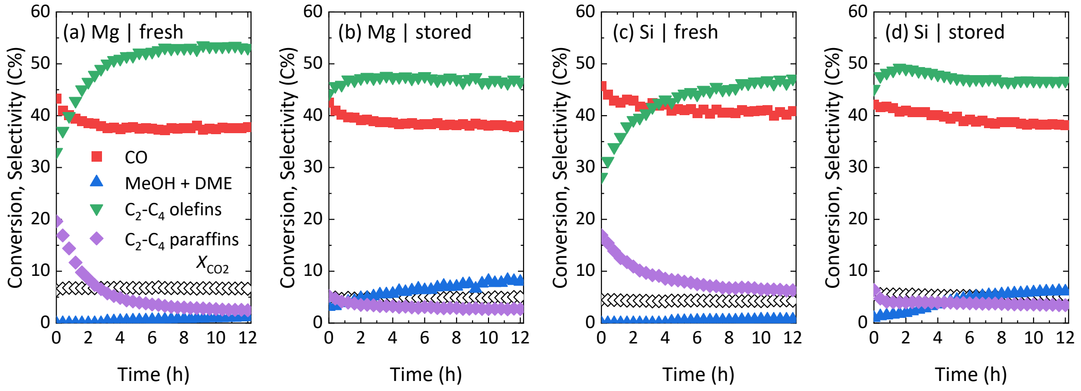

Figure S13. Evolution with time of CO_2 conversion and product distribution in the CO_2 tandem hydrogenation to hydrocarbons using ZnO:ZrO_2 catalyst + (a) freshly calcined MgAPO-18, (b) calcined and stored MgAPO-18, (c) freshly calcined SAPO-18 and (d) calcined and stored SAPO-18 in 1/1 catalyst ratio. 350 ºC, 30 bar, 12000 cm^3 h^{-1} g^{-1}, H_2/CO_2 ratio of 3.

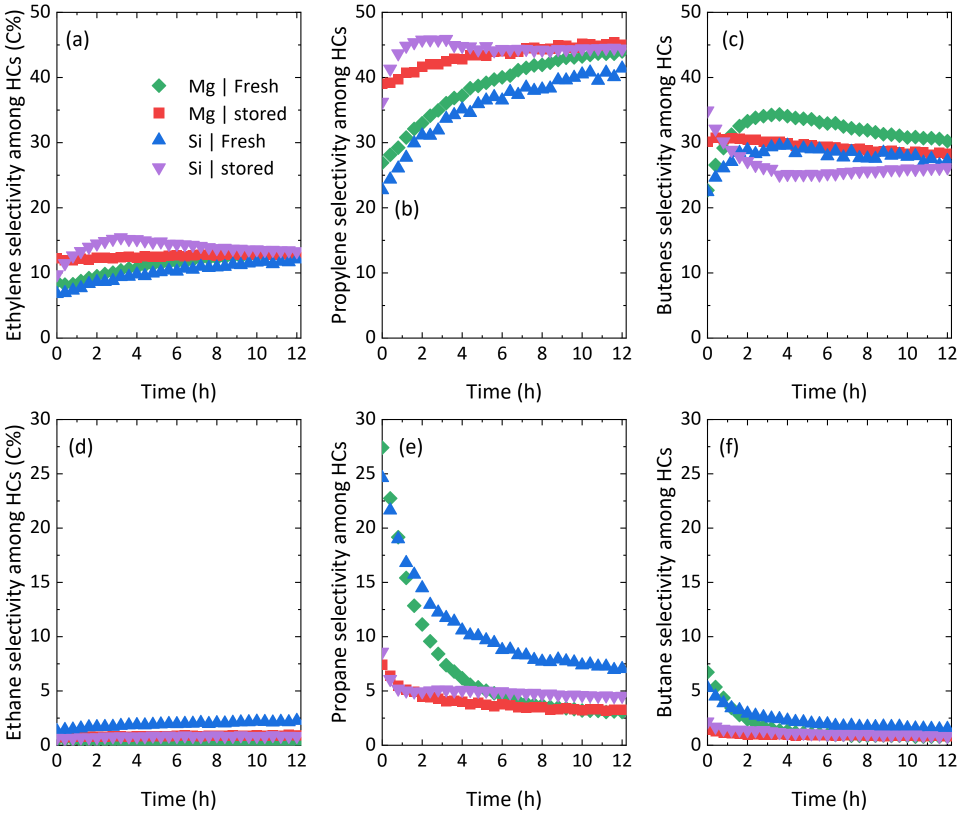

Figure S14. Evolution with time of hydrocarbon distribution in the CO_2 tandem hydrogenation to hydrocarbons using ZnO:ZrO_2 + MAPO-18 catalysts in 1/1 catalyst ratio. (a) Ethylene, (b) Propylene, (c) Butenes, (d) Ethane, (e) Propane and (f) Butane. 350 ºC, 30 bar, 12000 cm^3 h^{-1} g^{-1}, H_2/CO_2 ratio of 3.

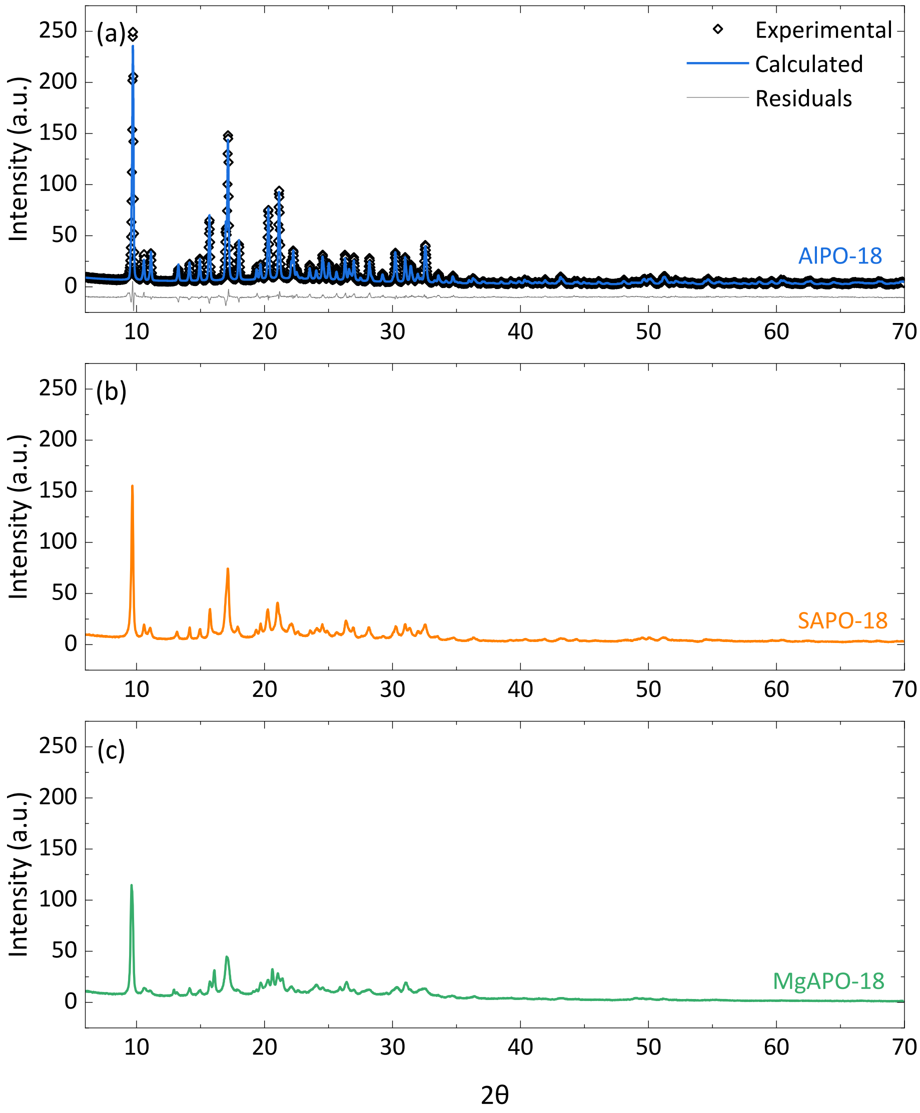

Figure S15. (a) Rietveld refinement of the as-made AlPO-18 zeotype, and experimental XRD diffractogram of the (b) SAPO-18 and (c) MgAPO-18 zeotypes.

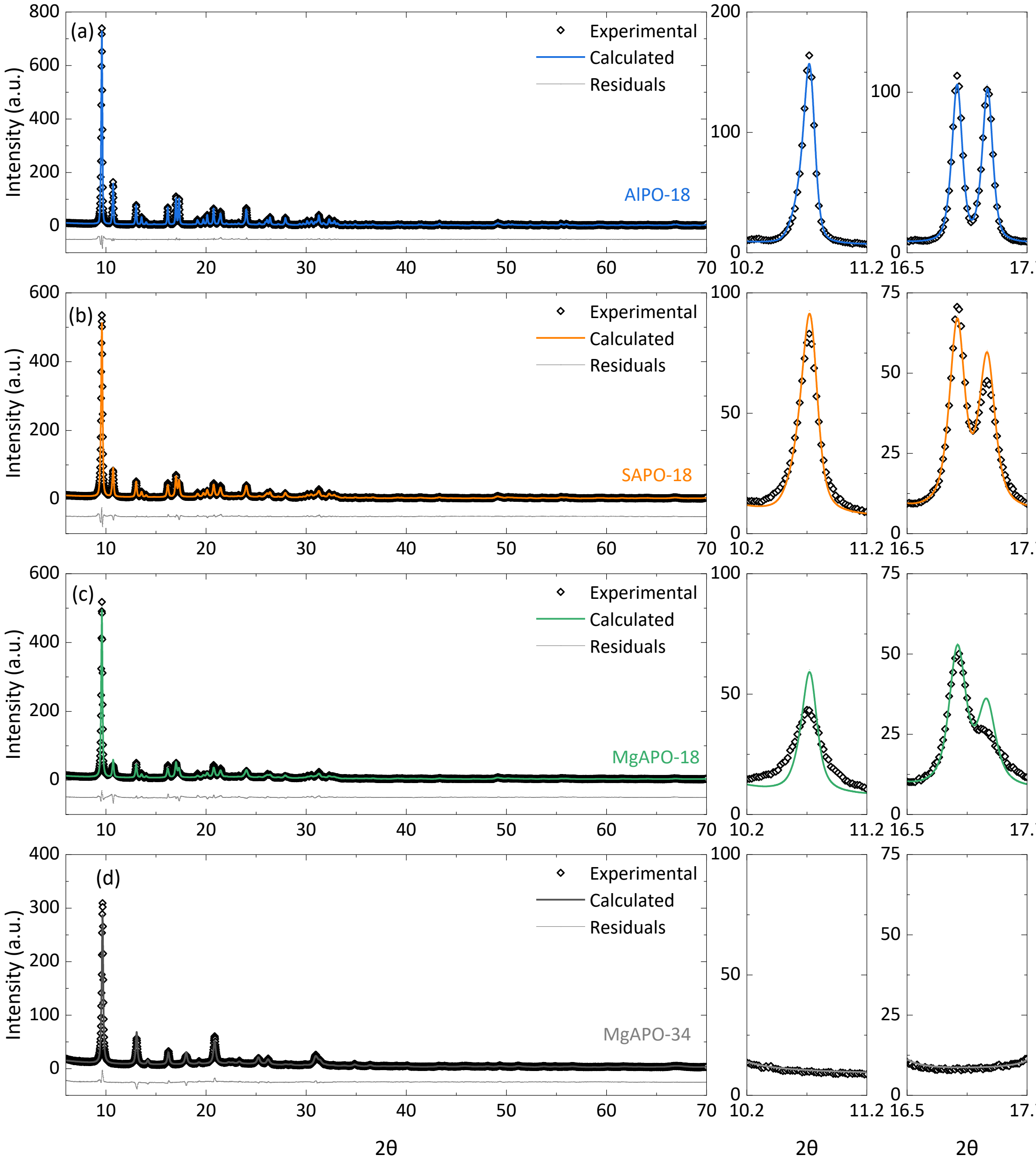

Figure S16. Rietveld refinement of the freshly calcined (a) AlPO-18, (b) SAPO-18, (c) MgAPO-18 and (d) MgAPO-34 zeotypes

Table S2. Summary table of Rietveld refined parameters.

	a (Å)	*b* (Å)	*c* (Å)	*b* (deg)	Unit cell vol ($Å^3$)	R_{wp} (%)
**AlPO-18**	**13.56**	**12.60**	**18.47**	**84.9**	**3144**	**9.8**
Evacuated	13.52	12.63	18.46	84.7	3139	7.8
Fresh cal	13.71	12.73	18.56	90.0	3237	8.0
Stored dry	13.71	12.73	18.56	90.0	3239	14.3
**SAPO-18**	**13.57**	**12.60**	**18.45**	**85.6**	**3145**	**11.7**
Fresh cal	13.71	12.73	18.57	90.2	3240	8.5
Stored dry	13.70	12.72	18.56	90.2	3233	7.0
**MgAPO-18**	**13.53**	**12.58**	**18.35**	**86.2**	**3115**	**16.3**
Fresh cal	13.69	12.71	18.58	90.2	3234	11.0
Stored dry	13.71	12.70	18.57	89.8	3234	5.7

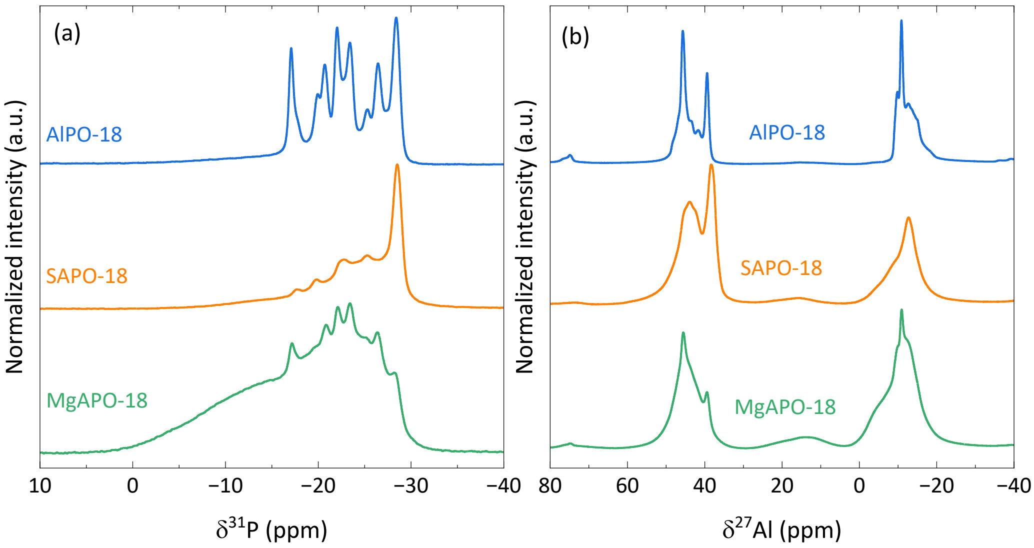

Figure S17. (a) ^{31}P and (b) ^{27}Al NMR of the stored (rehydrated) AlPO-18, SAPO-18, and MgAPO-18.

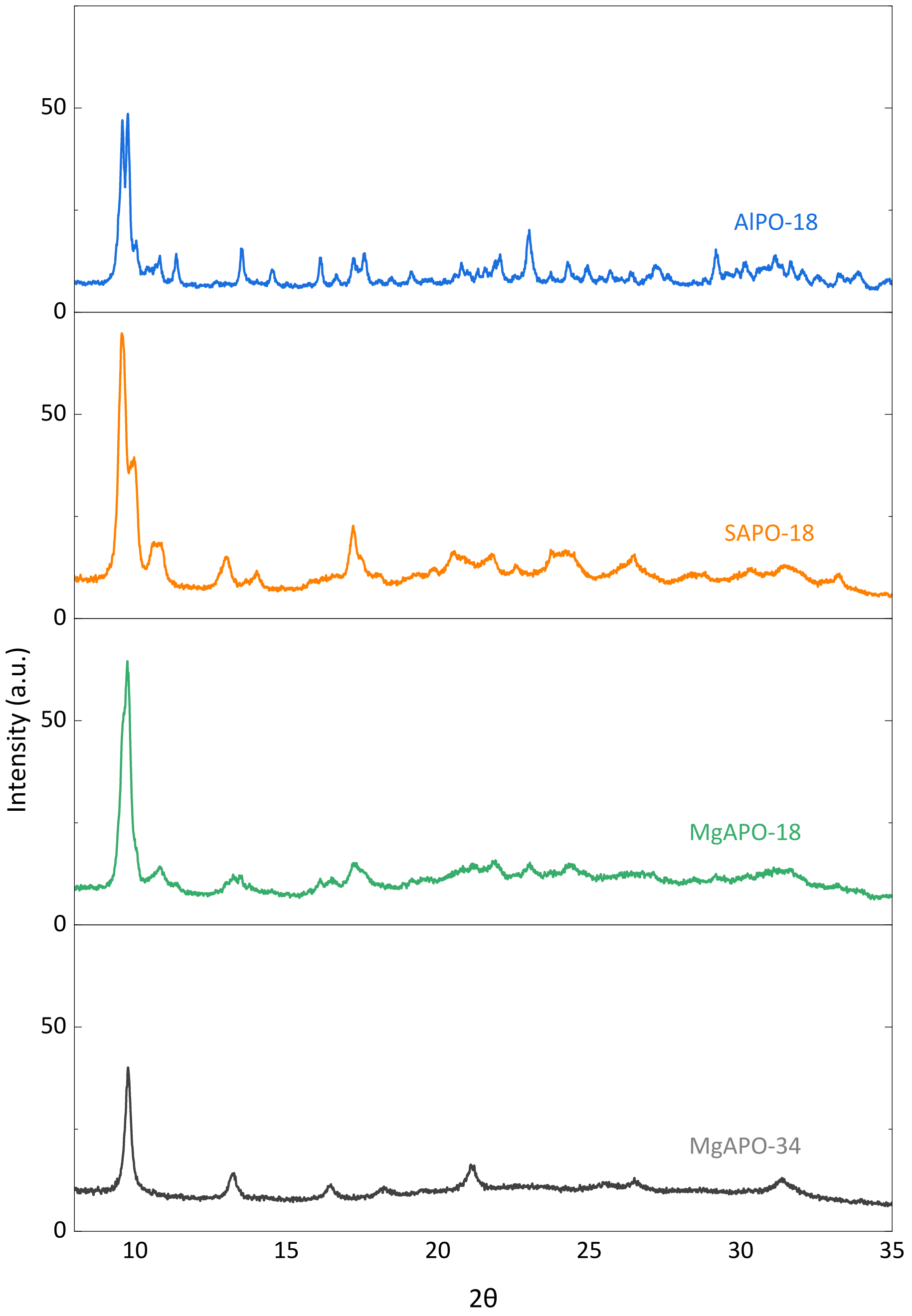

Figure S18. XRD diffractograms of the calcined and stored (rehydrated) AlPO-18, SAPO-18, MgAPO-18 and MgAPO-34.

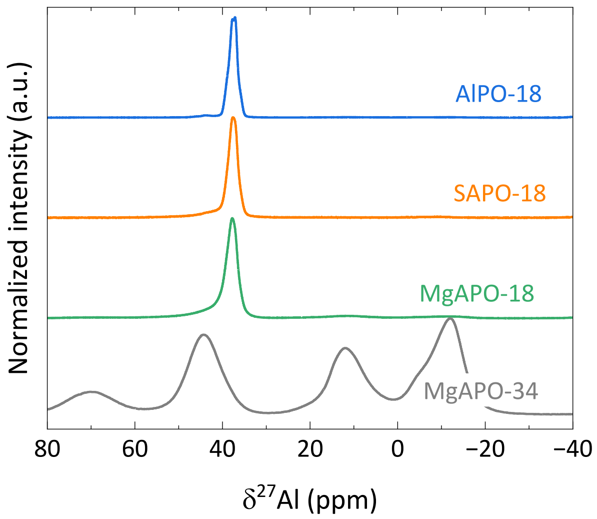

Figure S19. ^{27}Al NMR of the freshly calcined AlPO-18, SAPO-18, MgAPO-18 and MgAPO-34.

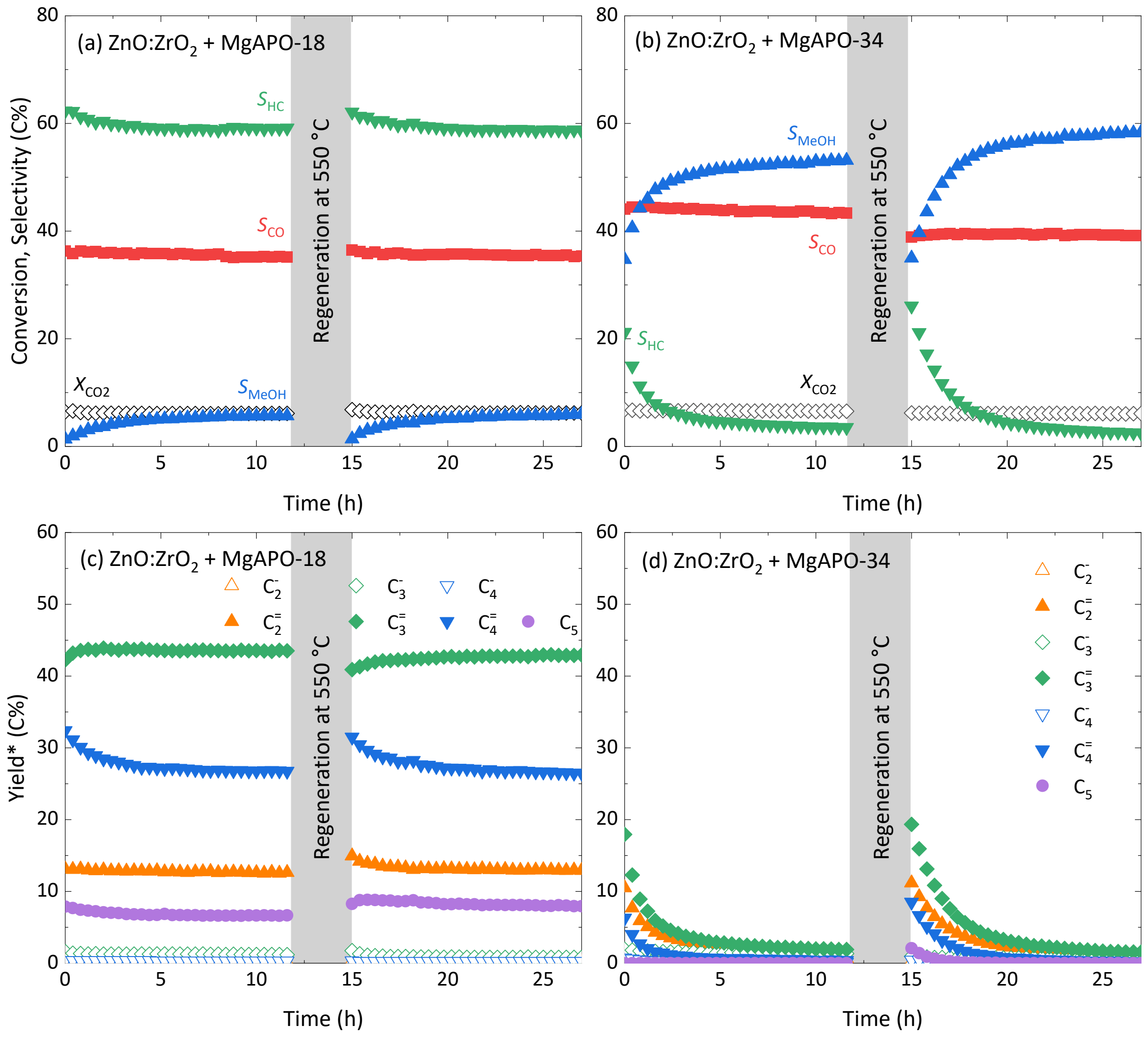

Figure S20. Evolution of (a, b) CO_2 conversion, CO, methanol and hydrocarbon selectivity and (c, d) hydrocarbon yields (recalculated on the basis of converted methanol) for two reaction cycles using ZnO:ZrO_2 catalyst + (a, c) calcined and stored MgAPO-18 and (b, d) freshly calcined MgAPO-34 in 1/1 catalyst ratio. 350 ºC, 30 bar, 12000 cm^3 h^{-1} g^{-1}, H_2/CO_2 ratio of 3.

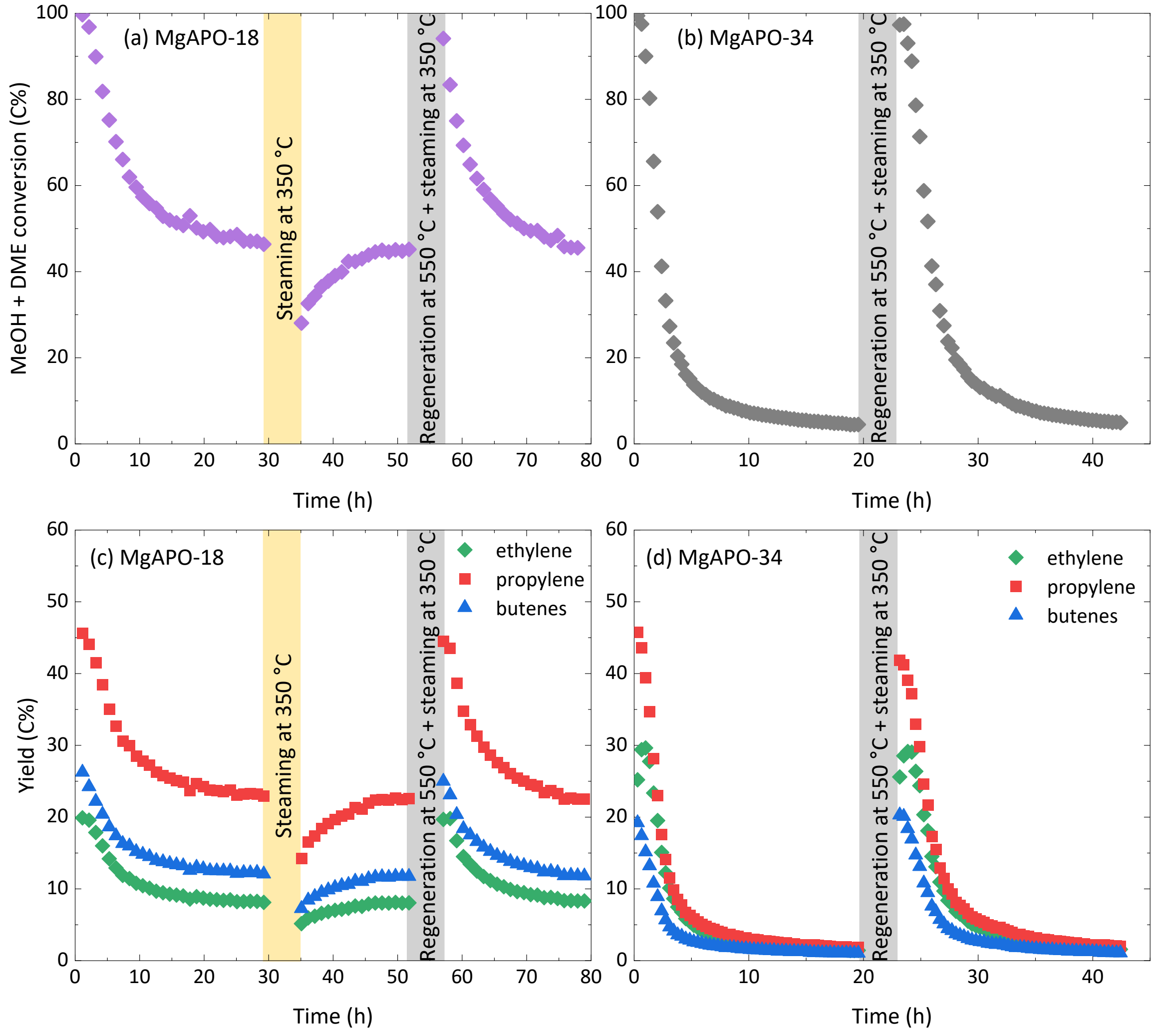

Figure S21. Evolution of (a, b) methanol + DME conversion (c, d) olefin yields for reaction cycles after subjecting the catalysts to steaming treatments at 350 ºC using the freshly calcined (a, c) MgAPO-18 and (b, d) MgAPO-34 catalysts. 350 ºC, 1 bar, 40 mbar MeOH in He, 3.7 g_{MeOH} h^{-1} g^{-1}.

Table S3. Textural and acid properties of the freshly calcined and calcined and stored MgAPO-18 zeotype.

	S_{BET} (m^2 g^{-1})	S_{ext} (m^2 g^{-1})	V_{mic} (cm^3 g^{-1})	V_{mes} (cm^3 g^{-1})	Density of acid site (mmol g^{-1})
MgAPO-18					
Fresh	742	61	0.26	0.70	0.46
Stored	297	35	0.10	0.42	0.31

Table S4. Textural properties of the freshly calcined MgAPO-18 and SAPO-18 and stored MgAPO-18 after water and methanol adsorption-desorption experiments at 25 ºC

	N_2 adsorption-desorption				CO_2 adsorption	
	S_{BET} (m^2 g^{-1})	S_{ext} (m^2 g^{-1})	V_{mic} (cm^3 g^{-1})	V_{mes} (cm^3 g^{-1})	S_{DR} (m^2 g^{-1})	V_{DR} (cm^3 g^{-1})
MgAPO-18						
Fresh	315	50	0.10	0.21	459	0.18
Stored	332	56	0.11	0.25	468	0.19
SAPO-18	668	74	0.23	0.70	903	0.36

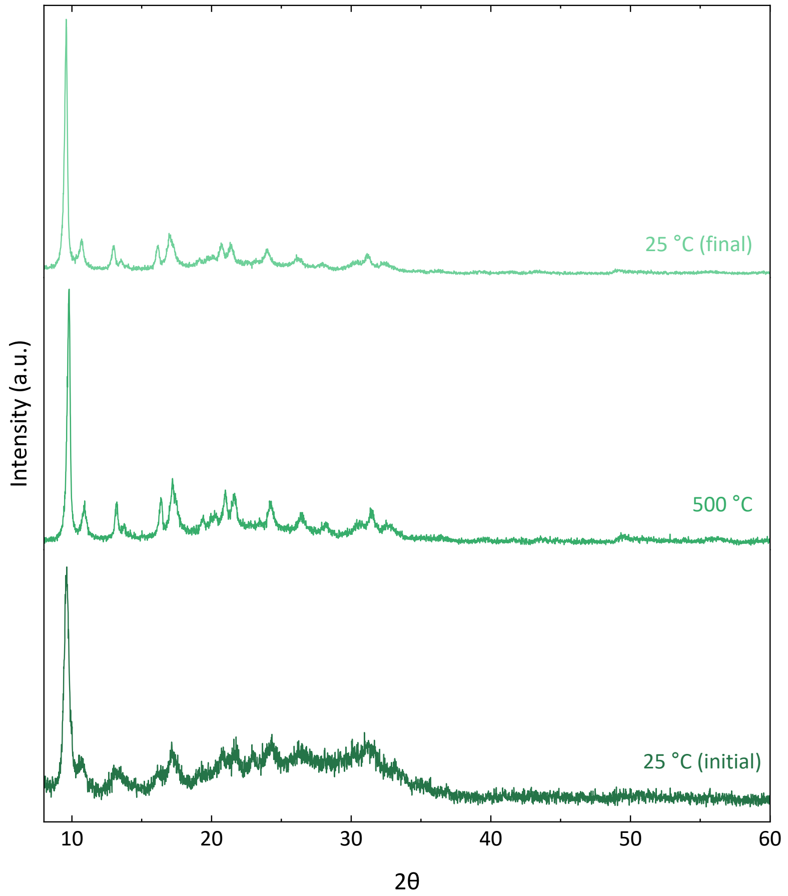

Figure S22. In-situ XRD diffractograms during a heat treatment after water adsorption-desorption experiments at 25 ºC for the freshly calcined MgAPO-18 zeotype

S4. Computational details

SAPO-18 and MgAPO-18 unit cells were generated from the AlPO-18 unit cell by substituting either a single Mg atom for Al or a single Si atom for P and including a charge-compensating proton to yield $Al_{23}P_{24}O_{96}MgH$ and $Al_{24}P_{23}O_{96}SiH$. The resulting Brønsted acid site (BAS) is in the same position for both unit cells (**Figure S23**).

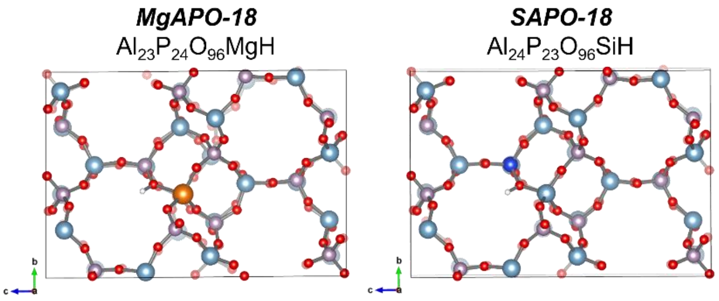

Figure S23. MgAPO-18 and SAPO-18 unit cells adopted for the computational simulations.

Following the full geometrical optimization of these unit cells at the DFT level of theory three surface species were generated and their IR spectra were simulated using the dipole-dipole autocorrelation function extracted from AIMD simulations: Brønsted acid sites, surface methoxy species and acetyl species. Moreover, two ketene derivatives inside the zeotype pores were simulated, namely dimethylketene, which has been experimentally identified within the induction of MTO in H-ZSM-513, and 6-fulvenone. To clarify the contribution of these guest species to the vibrational spectra computed from the overall unit cell, power spectra are presented which isolate the molecular modes from the zeolitic modes. The frequency associated with the C=O stretch appears at ca. 2085 and 2130 cm^{-1}, respectively, in our simulated power spectra. All infrared and power spectra of the surface and molecular species are illustrated in **Figure S24**.

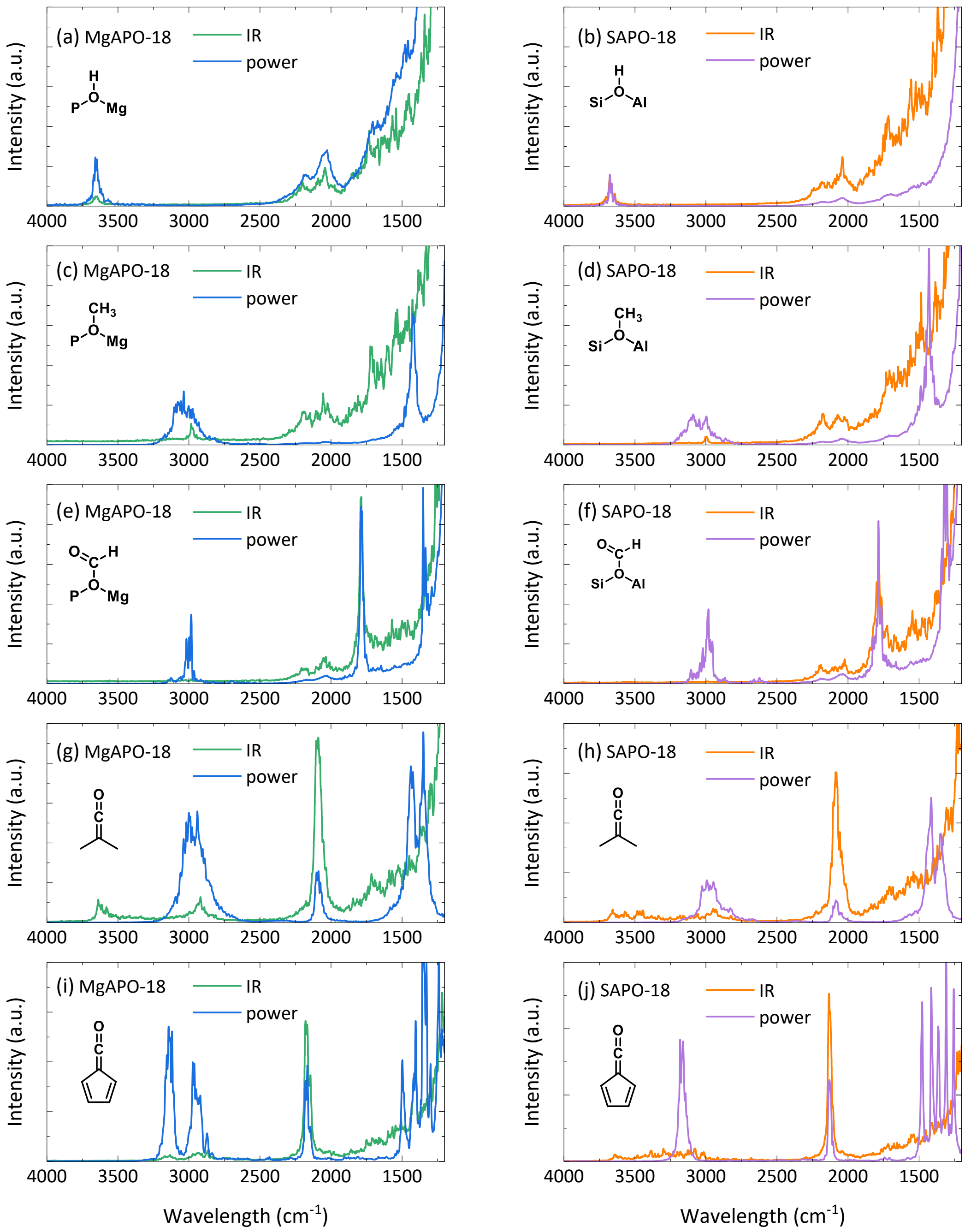

Figure S24. Simulated infrared and power spectra of the (a, b) Brønsted acid site, (c, d) surface methoxy species, (e, f) surface acetyl species, (g, h) dimethyl ketene and, (i, j) 6-fulvenone on the (a, c, e, g, i) MgAPO-18 and (b, d, f, h, j) SAPO-18 zeotypes.

For the 6-fulvenone ketene, the species was readily protonated by the Brønsted acid site in MgAPO-18 as shown in **Figure S25**, hence the reported spectra are of the cationic species rather than the neutral molecule. However, this proton transfer was not observed in the SAPO-

18 derivative, nor in the simulation of dimethylketene in either AlPO-18 derivative. Intermittent H-bonding interactions were also observed between the ketene carbonyl and Brønsted acid site in both models which resulted in some variation in the C=O stretch frequency.

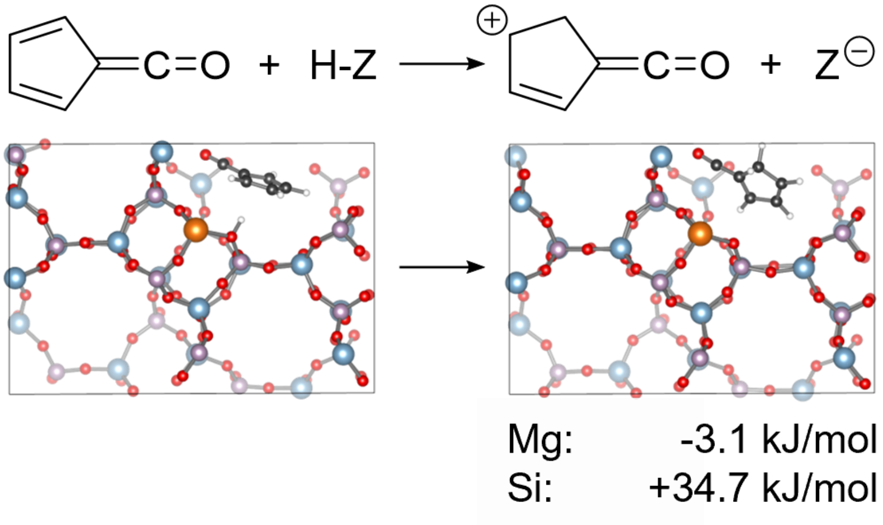

Figure S25. Protonation of 6-fulvenone by the BAS observed in the AIMD simulation of MgAPO-18, but not SAPO-18. The potential energy comparison confirms this proton transfer is favored for MgAPO-18 and disfavored in SAPO-18.

References

(1) Cnudde, P.; Redekop, E. A.; Dai, W.; Porcaro, N. G.; Waroquier, M.; Bordiga, S.; Hunger, M.; Li, L.; Olsbye, U.; Van Speybroeck, V. Experimental and Theoretical Evidence for the Promotional Effect of Acid Sites on the Diffusion of Alkenes through Small-Pore Zeolites. *Angew. Chemie - Int. Ed.* **2021**, *60*, 10016–10022. https://doi.org/10.1002/anie.202017025.

(2) Ramirez, A.; Gong, X.; Caglayan, M.; Nastase, S. A. F.; Abou-Hamad, E.; Gevers, L.; Cavallo, L.; Dutta Chowdhury, A.; Gascon, J. Selectivity Descriptors for the Direct Hydrogenation of CO2 to Hydrocarbons during Zeolite-Mediated Bifunctional Catalysis. *Nat. Commun.* **2021**, *12*, 5974. https://doi.org/10.1038/s41467-021-26090-5.

(3) Çağlayan, M.; Lucini Paioni, A.; Abou-Hamad, E.; Shterk, G.; Pustovarenko, A.; Baldus, M.; Chowdhury, A. D.; Gascon, J. Initial Carbon−Carbon Bond Formation during the Early Stages of Methane Dehydroaromatization. *Angew. Chemie - Int. Ed.* **2020**, *59*, 16741–16746. https://doi.org/10.1002/anie.202007283.

(4) Fu, D.; Lucini Paioni, A.; Lian, C.; van der Heijden, O.; Baldus, M.; Weckhuysen, B. M. Elucidating Zeolite Channel Geometry–Reaction Intermediate Relationships for the Methanol-to-Hydrocarbon Process. *Angew. Chemie - Int. Ed.* **2020**, *59*, 20024–20030. https://doi.org/10.1002/anie.202009139.

(5) Ticali, P.; Morandi, S.; Shterk, G.; Ould-Chikh, S.; Ramirez, A.; Gascon, J.; Chung, S. H.; Ruiz-Martinez, J.; Bordiga, S. PdZn/ZrO2+SAPO-34 Bifunctional Catalyst for CO2 Conversion: Further Insights by Spectroscopic Characterization. *Appl. Catal. A Gen.* **2023**, *655*, 119100. https://doi.org/10.1016/j.apcata.2023.119100.
